# Distinct processes contributing to post-submergence recovery determine leaf age-specific flood resilience in Arabidopsis

**DOI:** 10.1093/plphys/kiag317

**Published:** 2026-05-26

**Authors:** Tom Rankenberg, Maria Angelica Sanclemente, Hongtao Zhang, Hendrika A C F Leeggangers, Omar Orozco-Granados, Muthanna Biddanda Devaiah, Janet Rong Chao, Hans van Veen, Frederica L Theodoulou, Rashmi Sasidharan

**Affiliations:** Plant Stress Resilience, Utrecht University, Utrecht, the Netherlands; Plant Stress Resilience, Utrecht University, Utrecht, the Netherlands; Agricultural Sciences, Clemson University, Clemson, SC, United States; Vegetable Laboratory, US Department of Agriculture, Charleston, SC, United States; Rothamsted Research, Harpenden, United Kingdom; Plant Stress Resilience, Utrecht University, Utrecht, the Netherlands; Plant Stress Resilience, Utrecht University, Utrecht, the Netherlands; Experimental and Computational Plant Development, Utrecht University, Utrecht, the Netherlands; Plant Stress Resilience, Utrecht University, Utrecht, the Netherlands; Plant Stress Resilience, Utrecht University, Utrecht, the Netherlands; Groningen Institute for Evolutionary Life Sciences, University of Groningen, Groningen, the Netherlands; Rothamsted Research, Harpenden, United Kingdom; Plant Stress Resilience, Utrecht University, Utrecht, the Netherlands

## Abstract

While the molecular mechanisms mediating submergence tolerance have been extensively studied, those underpinning age-dependent resilience remain poorly characterized. In *Arabidopsis thaliana*, submergence elicits a leaf age–dependent phenotype in which senescence and death progress across an age gradient starting with older leaves. Here, we sought to investigate the mechanisms mediating the observed differential flood resilience by interrogating leaf age–specific transcriptome and proteome changes during submergence and recovery. Following submergence, most age-dependent differences were in the magnitude or speed of transcript abundance changes, whereas qualitative leaf age–dependent responses were most apparent during recovery. This included a strong desiccation response in old leaves despite a stronger abscisic acid (ABA)-signaling response. Physiological measurements suggested that faster dehydration was facilitated by submergence-mediated reduction of ABA sensitivity but was not associated with differences in stomatal conductance or cuticle integrity. We also observed a stronger induction in young shoot tissue of genes associated with endoplasmic reticulum stress and the unfolded protein response. Mutants disabled in both unfolded protein response signaling branches were affected in new leaf formation and in the ability to restore the proteome, but not in senescence, suggesting that young tissues activate endoplasmic reticulum stress recovery to permit continuation of growth. Among mitochondrial membrane proteins differentially regulated in young leaves, loss of mitochondrial voltage-dependent anion channel function impacted submergence-recovery tolerance. Our data reveal multiple mechanisms underlying leaf age–dependent differential submergence recovery and demonstrate how an interplay between age-related developmental traits and stress signaling pathways determines tolerance.

## Teaser

Leaf age–related developmental traits interact with stress signaling pathways to trigger distinct flood recovery mechanisms in Arabidopsis.

## Introduction

Complete submergence exposes the entire plant to stress (Sasidharan et al. 2017). However, the distinct identity of plant tissues can cause substantial differences in stress sensing and responses. This variation is not only a consequence of differences in physical properties but can also be due to differences in the molecular regulation of stress responses, ultimately leading to differential stress resilience (Rankenberg et al. 2021). Previous research has demonstrated both shared and unique responses to hypoxia and submergence stress between different cell types (Mustroph et al. 2009). Waterlogging induces the expression of cell wall–modifying enzymes, specifically in the root cortex leading to aerenchyma formation (Rajhi et al. 2011). Submergence can also elicit organ-specific responses (Van Veen et al. 2016). These studies compared cell types or organs that fulfill very different roles within a plant. However, variation in submergence responses can also be determined by plant age. In Arabidopsis, submergence tolerance declines during the transition from 2-wk-old seedlings to 5-wk-old adult plants. This decrease in tolerance stemmed from a reduction in the ability of ETHYLENE RESPONSE FACTOR GROUP VII (ERFVIIs) and the Arabidopsis NAC domain containing protein (ANAC) transcription factor (TF) ANAC017 to activate their downstream targets (Giuntoli et al. 2017; Bui et al. 2020). During submergence, the activity of the ERFVII RELATED TO APETALA 2.12 (RAP2.12) is limited by the trihelix TF HYPOXIA RESPONSE ATTENUATOR1 (HRA1) (Giuntoli et al. 2014). HRA1 binds to RAP2.12 and reduces its activity as a transcriptional activator, which attenuates the induction of anaerobic metabolism by RAP2.12. The repression of RAP2.12 activity by HRA1 is strongest in young leaves and meristems, where it likely serves to limit the utilization of carbon resources during hypoxia. Limiting carbon consumption during hypoxia is not only beneficial to preserve energy for post-stress recovery but also prevents premature activation of metabolic adjustments to hypoxia, since meristems are already hypoxic under non-flooded conditions (Weits et al. 2014).

These studies have thus clearly demonstrated how differential regulation of principal hypoxia response regulators such as ERFVIIs, HRA1, and ANAC017 leads to age-associated changes in submergence tolerance. The decrease in flooding tolerance with age is observed for both whole plants and individual leaves. Changes in stress tolerance of tissues with age can simply be a consequence of changes in physiology but can also be an actively regulated process. Several specific TFs were recently linked to changes in age-dependent stress tolerance (D’alessandro et al. 2018; Zhao et al. 2022), suggesting that age-dependent stress responses are often actively regulated at a molecular level.

A frequently observed pattern in age-dependent stress responses is a decrease in stress tolerance with leaf age (Rankenberg et al. 2021; Rankenberg et al. 2024). This can be caused by the inability of old leaves to properly respond to a stress (D’alessandro et al. 2018) or by the faster induction of stress-induced senescence in old leaves (Schippers 2015). Actively allowing an old leaf to die by accelerating the onset of senescence while a young leaf remains alive can be beneficial to a stressed plant, as nutrients can be transported from dying old tissues to young tissues and meristems to fuel their survival (Yu et al. 2015). Prioritizing the survival of more expendable tissues to keep meristems alive could permit plant reproductive success during stressful conditions. Despite the observed age dependence of stress tolerance in many species, the underlying molecular mechanisms are underexplored. Most studies tend to neglect the age dimension, focusing instead on bulk tissues where such differences might be lost. Capitalizing on the very clear age-directed senescence gradient induced in Arabidopsis rosettes upon complete submergence, we previously demonstrated that submergence triggers leaf age–independent activation of ethylene signaling via ETHYLENE-INSENSITIVE 3 (EIN3), but senescence is initiated only in old leaves. Although EIN3 stabilization leads to accumulation of the senescence-promoting TF ORESARA1 (ORE1) in both old and young leaves during submergence, leaf age–dependent senescence could be explained by ORE1 protein activation via phosphorylation specifically in old leaves (Rankenberg et al. 2024). Sequentially experiencing flooding followed by re-aeration stress results in a set of confounding effects determining the success of individual plant organs. How organ age and these confounding effects intersect to determine performance and what the limiting/causative factors are remain unclear.

To identify the processes and regulators that play a role in this age-dependent response to submergence stress, we characterized and compared the transcriptomes of an old leaf and a young leaf of Arabidopsis during and after complete submergence. Both the submergence and post-submergence phases triggered considerable changes in old and young leaf transcriptomes. Shared transcriptome responses to submergence between old and young leaves included the general repression of translation and growth. In old leaves, genes associated with leaf senescence exhibited increased expression during both the submergence and post-submergence phase. However, prolonged submergence treatment eventually also induced the expression of senescence-associated genes in young leaves. Although a strong correlation was observed between old and young leaf responses during the submergence phase, this was largely lost during recovery. The leaf age–dependent divergence of transcriptomic responses during recovery was consistent with the visual onset of stress between leaves during this phase. To further validate findings from the transcriptomics data we thus focused on leaf age–dependent processes evident during recovery: a strong desiccation transcriptome signature in old leaves and a stronger induction of endoplasmic reticulum (ER) stress genes in young leaves. Surprisingly, faster dehydration in old leaves could not be attributed to changes in ABA biosynthesis, stomatal density, aperture, conductance, or cuticle thickness but instead was associated with a submergence-mediated decrease in ABA sensitivity in these leaves. Consistent with our transcriptomics findings, ER stress sensing and signaling is important for submergence recovery. Mutants for components of ER stress sensing were compromised in submergence survival. Importantly, mutant phenotypes deviated in terms of “young leaf” attributes, that is new leaf formation and not submergence-induced senescence. ER stress-responsive genes were also preferentially induced in young leaves during recovery. This was confirmed by comparison of the transcriptome and proteomes of mutants disabled in ER stress sensing and signaling. This global assessment also revealed the preferential upregulation in young leaves of proteins contributing to maintenance of cellular homeostasis and faster recovery. This included a group of mitochondrial proteins, the voltage-dependent anion channels, that have recently been implicated in mediating mitophagy (Ma et al. 2025). Overall, our data provide a comprehensive insight into molecular pathways mediating leaf age–dependent differential submergence sensing and recovery.

## Results

### Submerged Arabidopsis rosettes exhibit age-dependent sequential leaf death

**Figure 1:**
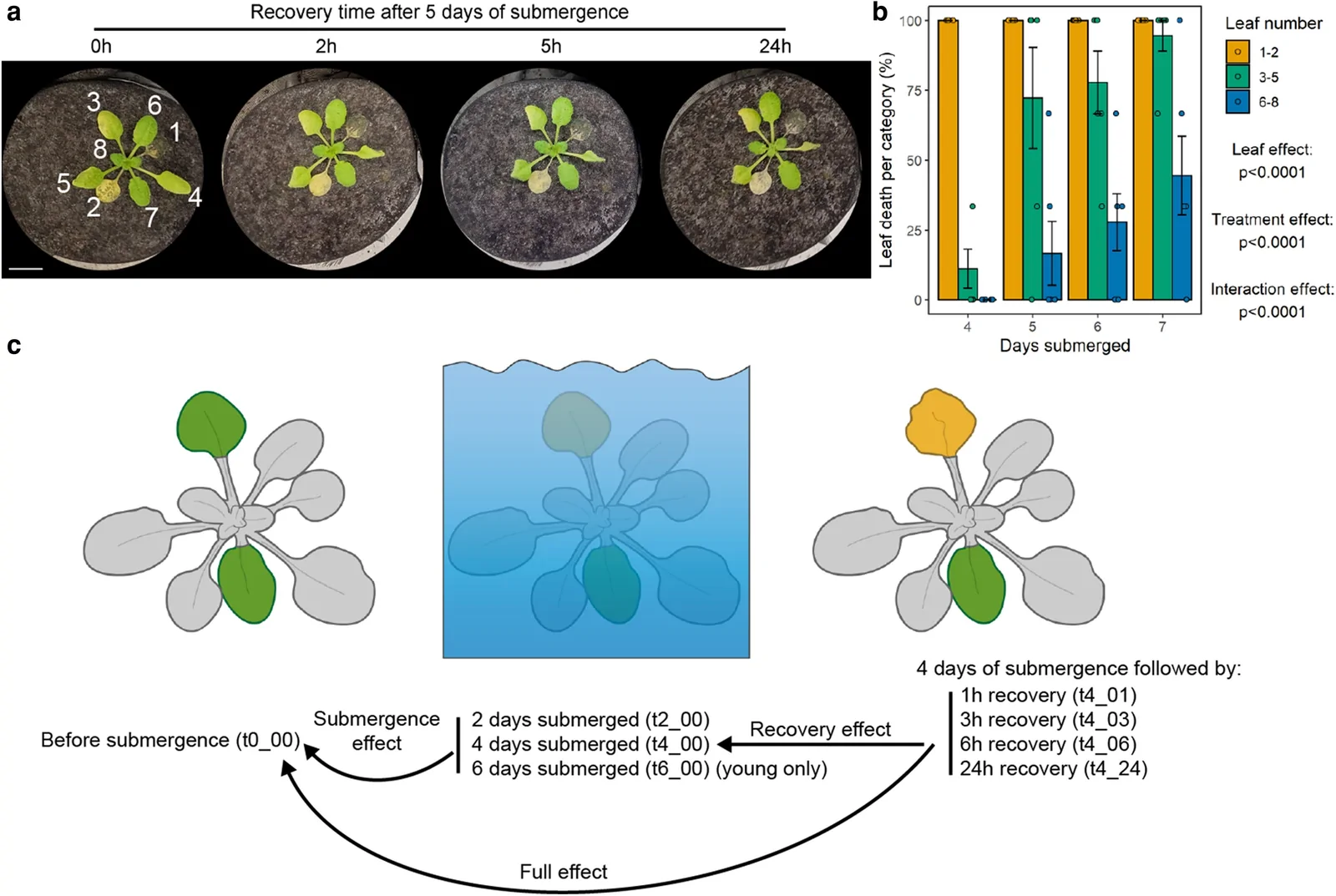
Resilience to the submergence and post-submergence stress decreases with leaf age. a) Representative images of wild-type Arabidopsis plants (accession Col-0) recovering from 5 d of dark submergence and showing age-dependent leaf damage and death. Images were taken at the recovery time points indicated. Numbers in the first image (0 h recovery) indicate leaf numbers. Scale bar indicates 1 cm. Images were digitally extracted for comparison. b) Submergence causes age-dependent leaf death. Leaf death per age category was scored 3 d after de-submergence after the indicated submergence durations. Values are mean ± SE. n = 6 plants per treatment. Significance was determined by 2-way ANOVA (leaf age*time). Leaf numbers are as indicated in Fig. 1a. c) Overview of the mRNA-seq experimental design and treatment comparisons. Laminas of old (leaf 3) and young (leaf 7) leaves of 10-leaf-stage Arabidopsis plants were harvested before submergence and after the indicated treatments. Gene expression was calculated for each submergence timepoint relative to non-submerged conditions (submergence effect), for the post-submergence timepoints relative to the last submerged timepoint before de-submergence (recovery effect), and for the post-submergence timepoints relative to the non-submerged conditions (full effect).

Complete submergence in darkness (hereafter submergence) of Arabidopsis Col-0 plants for varying durations triggered a sequential leaf death pattern (Fig. 1a). Visible senescence and leaf death was initiated in the oldest leaves and progressed toward younger leaves, consistent with our previous observations (Rankenberg et al. 2024). Plants recovering from sublethal submergence treatments showed further deterioration following de-submergence, with senescence and leaf dehydration in the post-submergence phase being more severe in old and intermediate leaves than in young leaves. The age-dependent effects of the submergence and post-submergence phases led to age-dependent leaf death: the old leaves died faster than the young leaves (Fig. 1b). To characterize the molecular responses and potential regulatory genes and processes underlying this differential age effect, a mRNA-seq approach was used. Blades of leaf 3 and 7 of a 10-leaf Arabidopsis plant (Fig. 1a) were sampled at 8 timepoints (Fig. 1c). Leaf 3 was chosen as a representative “old” leaf. It resembles most other rosette leaves, apart from leaves 1 and 2, which have a rounder shape and die faster than the rest (Fig. 1c). Leaf number 7 was the representative “young” leaf. It is the youngest leaf at this growth stage that has an easily distinguishable petiole. Despite their physiological similarities, these 2 leaves died at different rates following submergence (Fig. 1b). Sampling for mRNA-seq occurred before submergence (t0_00) and after 2 (t2_00), 4 (t4_00), and 6 (t6_00) d of submergence. Leaf samples were also harvested after 4 d of submergence at 1 (t4_01), 3 (t4_03), 6 (t4_06), and 24 (t4_24) h in the light after de-submergence (Fig. 1c). Only the young leaf was sampled at the 6 d submergence timepoint, as the old leaf was often dead at this time. The submergence treatment used here arrests developmental leaf progression and submerged plants do not make new leaves underwater. The 10-leaf-stage plants (t0_00) are therefore the most suitable developmentally matched controls for assessing submergence effects compared with air-grown plants. Differential gene expression was calculated at: (1) each submergence timepoint compared with the non-submerged plants (the submergence effect); (2) each post-submergence phase time point relative to 4-d submerged plants (the recovery effect); (3) each post-submergence timepoint compared with the non-submerged plants (the full effect). The first 2 approaches allowed us to investigate the specific response to the submergence and post-submergence phases without including the effect of the sequential exposure to these phases.

### The submergence and post-submergence phases together have a stronger effect on the transcriptome of old leaves

**Figure 2:**
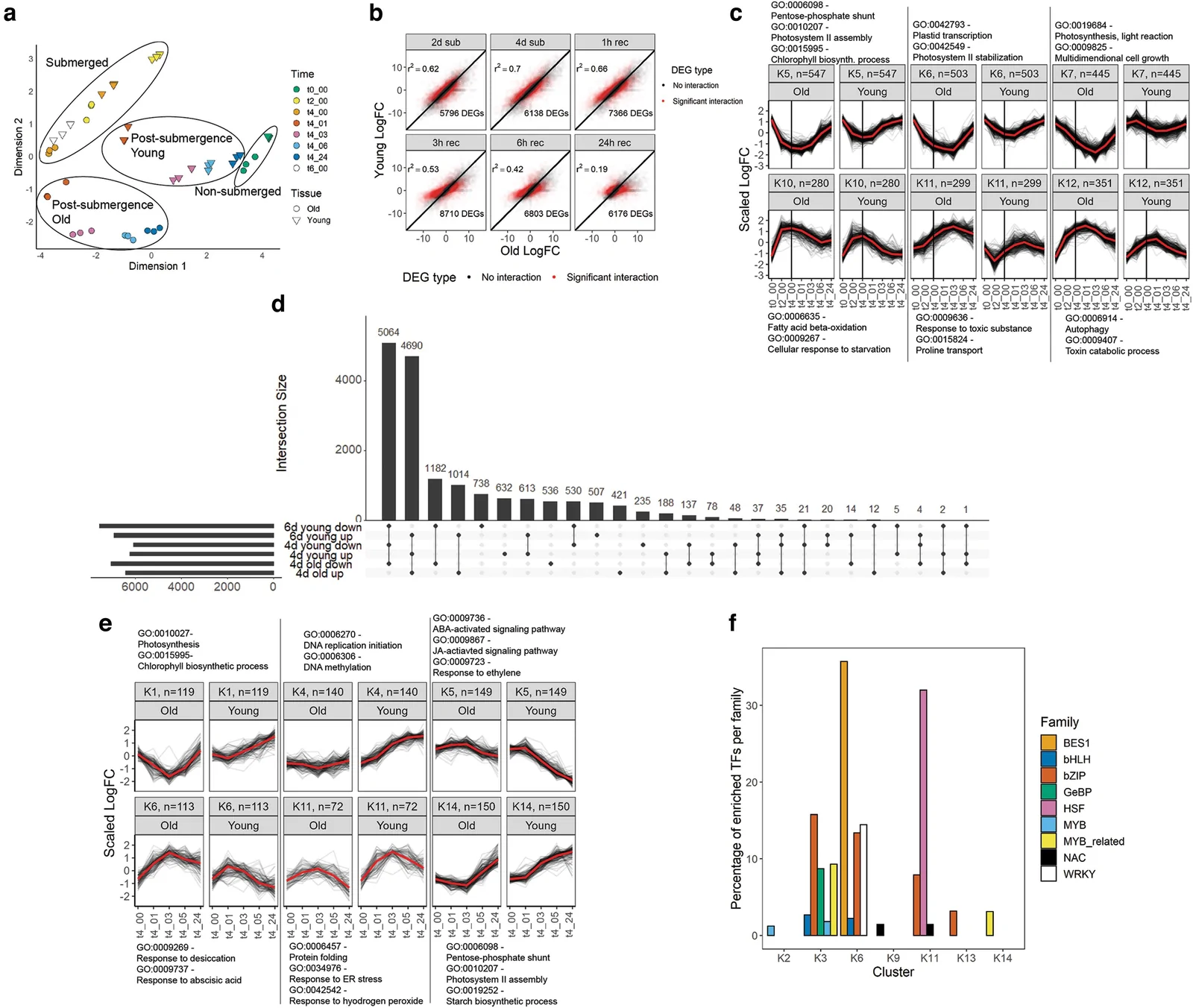
Submergence and recovery both induce a leaf age-dependent reconfiguration of the transcriptome. a) Multidimensional scaling (MDS) plot showing the distribution of the 3 biological replicates of the control (t0_00), submergence (t2_00, t4_00), and post submergence (t4_00, t4_01, t4_03, t4_06, t4_24) old and young leaf samples. b) Scatterplots comparing the response (log2FC) of individual genes in old and young leaves for the various submergence and recovery time points (20,588 genes). Transcripts with a significant interaction for leaf age*time are highlighted in red, and the number of these DEGs is shown in the bottom-right of each panel. Pearson r^2^ of the correlation between gene expression in old and young leaves in each timepoint is shown in the top-left of each figure. DEGs were calculated based on n = 3. c) Clustering of the 4,977 genes with significantly different expression between old and young leaves at a minimum of 4 timepoints across all sampled timepoints. Expression was calculated for old and young leaves relative to their respective expression under control conditions. A selection of overrepresented GO terms are shown per cluster. d) UpSet plot (input 5,485 DEGs) showing the intersection between genes induced or repressed after 4 or 6 d of submergence in young leaves and genes induced or repressed after 4 d of submergence in old leaves. Dots show which categories are intersected, numbers above each vertical bar indicate the size of each intersect. Horizontal bars indicate the size of each individual category. DEGs were calculated based on n = 3. e) Clustering of the 1,845 genes with significantly different expression between old and young leaves at a minimum of 2 timepoints across all post-submergence timepoints. Expression was calculated for old and young leaves relative to their respective expression at the last timepoint before de-submergence. A selection of overrepresented GO terms is shown per cluster. f) The percentage of transcription factors per family of which the targets are significantly enriched among the genes in clusters from Fig. 2e and Fig. S5. Sample size for testing target enrichment 28,643 genes. DEGs were calculated based on n = 3.

Multidimensional scaling (MDS) of the 2,000 most variable genes showed that transcriptomes of old and young leaves were relatively similar before submergence (t0_00, Fig. 2a). However, the submergence time points (t2_00, t4_00, and t6_00) all clustered far away from the pre-submergence time points, suggesting massive transcriptional reprogramming during submergence in both old (circles) and young leaves (triangles). The post-submergence time points (t4_01, t4_03, t4_06, and t4_24) of old leaves revealed a second phase of transcriptional reprogramming. At 24 h after de-submergence, old leaf transcriptomes were still far removed from the t0_00 time point. This was in contrast with the pattern of young leaf post-submergence timepoints, where the last post-submergence timepoint clustered very close to the t0_00 timepoint, suggesting more rapid recovery. The number of differentially expressed genes (DEGs) detected for old and young leaves was consistent with the MDS plots. DEG numbers were similar at submergence time points but diverged during recovery with lower numbers in young than in old leaves (Fig. S1a; Table S1). These patterns were consistent with observed leaf phenotypes after the post-submergence phase: the old leaf was generally barely alive and severely damaged, whereas the young leaf fully recovered and continued to grow. Since recovery effects were based on comparisons of the 4 recovery time points to a single submergence time point, it is possible that some of the detected transcript expression patterns observed are attributable to circadian (time-of-day) variation. To investigate this possibility, we picked a set of classic circadian clock marker genes (Michael et al. 2008) and compared their reported circadian/diurnal expression profiles with those observed in our dataset (Fig. S2). The rhythmic amplitudes of these genes were markedly dampened in our experiments relative to these published time courses. So, although some contribution of circadian variation cannot be excluded, these comparisons suggest it is unlikely to be a major driver of the recovery-associated transcriptome changes reported here.

To analyze leaf age-dependent changes in gene expression, we also calculated DEGs between old and young leaves. At each of the 6 sampling timepoints during the submergence and post-submergence phases, more than 5,000 genes showed a leaf age-dependent response to the treatment (Fig. S1b). This number was highest at the 3-h post-submergence timepoint, where 8,710 genes showed an age-dependent response. Although many genes responded in a leaf age-dependent manner to submergence, the correlation in gene expression between old and young leaves was relatively high during this phase (Fig. 2b). This correlation was largely lost during the post-submergence phase, indicating an age-dependent divergence of the transcriptomes during this recovery phase.

### Old and young leaves share some common responses to submergence and recovery

To explore the age-independent response to submergence, we first focused on the common stress responses between old and young leaves. We did this by selecting all genes whose expression was significantly different from t0_00 at 2 or more timepoints of the full time course (submergence and post-submergence), and selecting the genes in which the difference in response between old and young leaves was never significant (an FDR-adjusted *P* > 0.05 and/or an absolute log_2_ fold change < 1). This approach led to a selection of genes that change in expression during the submergence and post-submergence treatments, but in an age-independent way. The resulting list of 1,629 genes was then divided over 6 clusters by fuzzy k-means clustering, which allowed us to identify common patterns in gene expression (Fig. S3a). Genes with a maximum cluster membership lower than 0.50 were then omitted to reduce the noise in the dataset. The remaining 1,482 genes, whose response to the treatments was independent from leaf age, were then further analyzed by testing which gene ontology (GO) terms were enriched per cluster (Fig. S3b).

Several of the GO terms significantly enriched in cluster K1 were related to immune responses, like GO:0010200 “response to chitin” and GO:0002679 “respiratory burst involved in defense response”. Immune responses are known to be induced by submergence treatment (Hsu et al. 2013). This was consistent with the expression pattern of genes in cluster K1, which were gradually upregulated during submergence and slightly further upregulated during the post-submergence phase. Cluster K1 was also enriched for genes associated with endoplasmic reticulum stress, like those in categories GO:0034976 “response to endoplasmic reticulum stress” and GO:0006457 “protein folding”. Lastly, cluster K1 was enriched for genes associated with ethylene-related GO terms, like GO:0009723 “Response to ethylene”. The age-independent response to ethylene was likely a result of systemic accumulation of ethylene in submerged plants (Voesenek and Sasidharan 2013; Sasidharan and Voesenek 2015).

Many of the GO terms enriched in cluster K2 were related to growth and photosynthesis, processes that are paused during submergence and reactivated during the post-submergence phase. This pattern of downregulation during submergence and reactivation during the post-submergence phase was also found in cluster K6, although the downregulation was much steeper here. Cluster K6 was enriched for GO terms involved in translation, consistent with previous reports that have found a rapid general shutdown of this process during low-oxygen stress, although translation of specific transcripts is increased (Sachs et al. 1980; Juntawong et al. 2014; Cho et al. 2022). Clusters K2 and K6 were the 2 gene clusters with the most members (282 and 280 members, respectively), which highlights how common the shutdown of processes associated with growth and development was during submergence stress.

Surprisingly, we did not observe the enrichment of GO terms associated with hypoxia stress, such as GO:0001666 (“Response to hypoxia”). The expression of multiple core hypoxia genes varied considerably between old and young leaves, although there was no trend toward a stronger response in either leaf during submergence (Fig. S4). During the post-submergence phase, however, expression of core hypoxia genes was reduced faster in young leaves than in old leaves (Fig. S4).

### Age-dependent leaf responses to submergence and recovery

To identify the leaf age–dependent responses with a potential role in stress tolerance, genes that showed a significant age-dependent response to submergence or recovery at a minimum of 4 out of the 6 timepoints were selected. These were visualized by classification into 14 clusters by fuzzy k-means clustering, omitting the genes with a maximum cluster membership lower than 20% to reduce noise (Fig. S5a, Fig. 2c). This revealed that the pausing of photosynthesis and growth during the submergence phase occurred more rapidly in old leaves than young leaves, as GO terms “Leaf morphogenesis”, “Plastid transcription”, and “Photosynthesis, light reaction” were overrepresented in clusters K5-K7 (Fig. S5b, Fig. 2c). These processes were not only arrested faster in old leaves during the submergence phase, but they also resumed more slowly during the post-submergence phase. As expected, the induction of genes associated with senescence-related GO categories was stronger in old leaves than in young leaves (clusters K10 and K12) concomitant with the earlier initiation of senescence in older leaves (Rankenberg et al. 2024). Measurement for the enrichment of targets of TFs per cluster revealed candidates regulating age-dependent responses. Of note was identification of clusters strongly enriched for targets of WRKY DNA BINDING PROTEINS (WRKYs) (clusters K2 and K13) and BASIC LEUCINE ZIPPERs (bZIPs) (Cluster K6) TFs (Fig. S5c).

For all sampling timepoints (Fig. 1c), both an old leaf and a young leaf were harvested. The exception was the 6-d submergence timepoint, where only the young leaf was sampled since the old leaf was generally dead after 6 d of submergence. It was of interest to establish whether the transcriptome of a “near-death” young leaf after 6 d of submergence was like that of a “near-death” old leaf after 4 d of submergence. For this, the young leaf submergence effect DEGs after 4 d (6,246 upregulated and 6,094 downregulated genes) and 6 d (6,934 upregulated and 7,555 downregulated) of submergence were overlapped with the old leaf submergence effect DEGs after 4 d of submergence (6,431 upregulated and 7,053 downregulated). The majority of DEGs analyzed were significantly down- or upregulated in all 3 of these sets of genes (Fig. 2d). Of the genes that were not shared between all 3 sets (3,652 for upregulated DEGs, 3,635 for downregulated DEGs), most overlap was between the transcriptomes of old leaves of plants submerged for 4 d and young leaves of plants submerged for 6 d, with 1,182 shared downregulated genes and 1,014 shared upregulated genes, respectively. This was also consistent with the MDS plot in Fig. 2a, in which the transcriptomes of old leaves submerged for 4 d and young leaves submerged for 6 d were located close to each other. Gene ontology analysis of these 2 sets of genes showed that processes commonly downregulated were often involved in fundamental cellular processes like transcription and translation (Fig. S6). Shared upregulated genes between old and young leaves at their last submergence timepoint were involved in transport-related processes, suggesting export of the last nutrients out of the dying leaf for use in other parts of the plant. Consistent with this, we found that prolonged submergence causes an induction of transcripts associated with autophagy (GO:0006914), leaf senescence (GO:0010150), and chlorophyll catabolism (Li et al. 2016) in both old and young leaves (Fig. S7). The high degree of similarity between the “near-death” transcriptomes of old and young leaves showed that a prolonged submergence treatment eventually had the same transcriptomic result in these leaves. The difference between the 2 leaves was in the time it took for them to reach this point.

### Age-dependent regulation of transcriptional responses during recovery

Clustering genes based on their expression patterns during the submergence and post-submergence phases allowed us to identify processes that are regulated in a leaf age–dependent manner. However, the dominant effect of the submergence phase might mask any age-dependent regulation that is specific to the post-submergence phase. To identify leaf age–dependent processes specific to the post-submergence phase, DEGs with a significant interaction effect for time*age at a minimum of 3 out of the 4 post-submergence timepoints were selected. These 1,848 genes were then divided over 14 clusters by fuzzy k-means clustering, and genes with a maximum cluster membership below 20% were again removed (Fig. S8). Consistent with the results in Fig. 2c, the increase in transcripts of genes related to photosynthesis and growth was faster during recovery in young than in old leaves (Fig. 2e clusters K1, K4, and K14, Figs. S8a and S8b). Cluster K5 showed that processes controlled by the stress-related hormones ethylene, ABA, jasmonic acid (JA), and salicylic acid (SA) were shut down faster in young leaves than old leaves during the post-submergence phase.

Clustering of the age-dependent responses to the recovery phase revealed a stronger and more sustained induction of genes associated with the GO categories “response to desiccation” and “response to abscisic acid” in old leaves (Fig. 2e, cluster K6). This was consistent with the visual phenotype of plants recovering from submergence, where there was more desiccation in old than in young leaves (Fig. 1a). ABA-mediated responses to dehydration are controlled by ABA-RESPONSIVE ELEMENT BINDING FACTOR (ABF) TFs of the bZIP family (Uno et al. 2000). The targets of several ABFs and other bZIP TFs were overrepresented among the genes in cluster K6 (Fig. 2f).

Cluster K11 showed a strong enrichment for multiple GO categories associated with protein misfolding and exposure to hydrogen peroxide, two processes that are often connected (Ozgur et al. 2018). Expression of genes in this cluster increased further in young leaves than in old leaves during the post-submergence phase. Targets of HEAT SHOCK FACTOR (HSF) and bZIP TFs were overrepresented in cluster K11 of genes responding to the post-submergence phase, which was consistent with the involvement of TFs of these families in these processes (Fig. 2f) (Jung et al. 2013; Ko and Brandizzi 2022). Of the HSF TFs whose targets are overrepresented in cluster K11, some had clear leaf age–dependent patterns (Fig. S9). The expression of both *HSFA6A* and *HSFA6B* was higher in young leaves than in old leaves during the first hours post-submergence. These 2 closely related TFs play a role in the interaction between ABA signaling and reactive oxygen species (ROS) signaling (Wenjing et al. 2020). Their higher expression in young leaves could facilitate their faster response to de-submergence, as ABA and ROS signaling are both important during submergence recovery (Yeung et al. 2019). In maize, the expression of a homolog of Arabidopsis *HSFA6B*, *HSFTF13* is induced by bZIP60 during heat stress (Li et al. 2020). Under heat stress, HSFTF13 induces the expression of HEAT SHOCK PROTEINS (HSPs), which function as chaperones for accumulated misfolded proteins under stress conditions. This is also consistent with the overrepresentation of genes associated with ER stress and protein folding in cluster K11 (Fig. 2e; Fig. S8).

### Age-dependent leaf dehydration during recovery can be attributed to differential ABA sensitivity but not stomatal conductance

**Figure 3:**
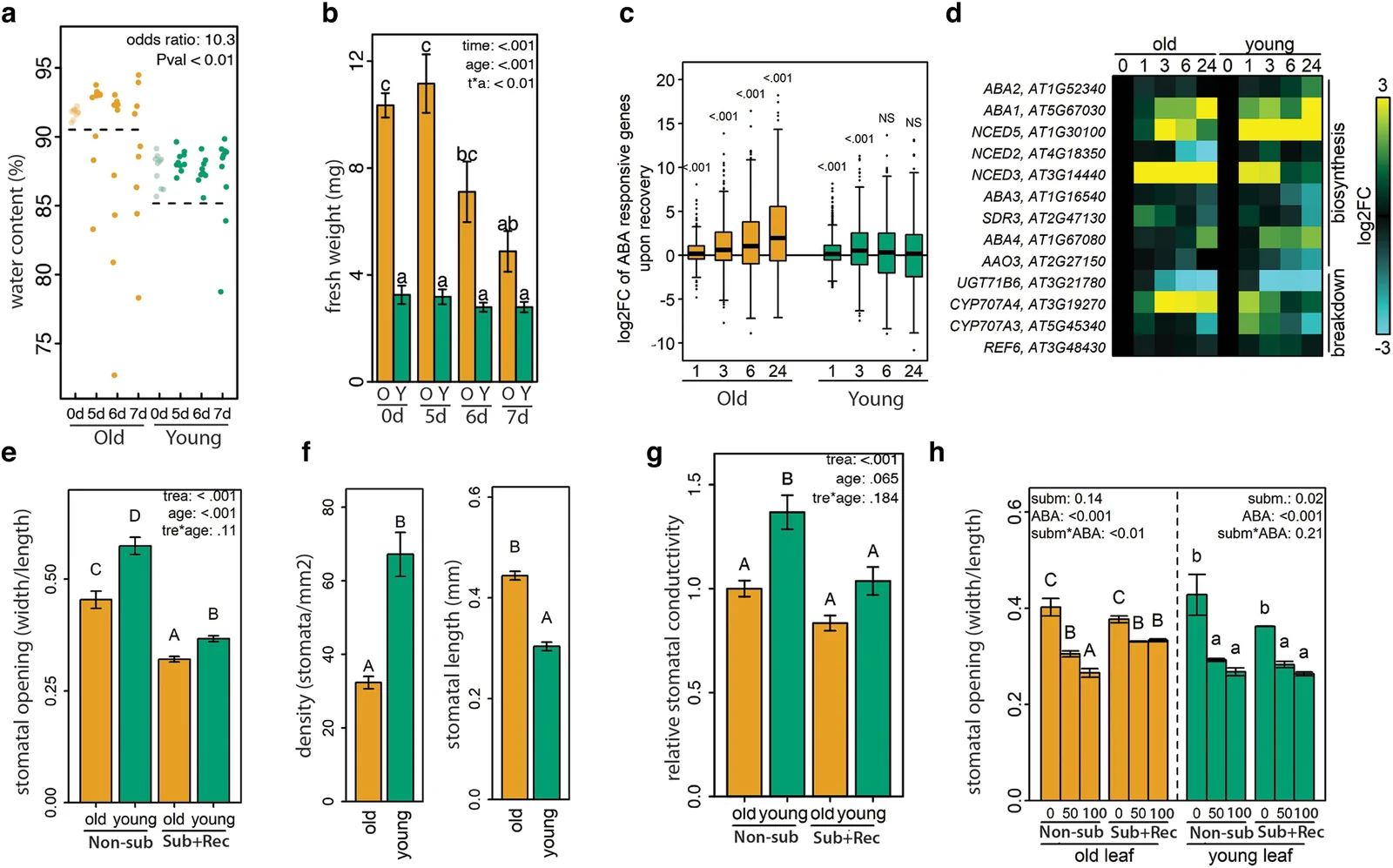
Differential ABA sensitivity and stomatal conductivity contributions to age-dependent dehydration during post-submergence recovery. a) Water content (the percentage of water per leaf weight) of old (orange) and young (green) leaves 1 d after the indicated submergence durations (in days [d]). Darker orange and green dots represent old and young leaves of submerged plants (respectively). The paler color represents leaves from the non-submerged plants. Leaves were considered to have lost the ability to maintain water relations if their water content dropped below the lowest values found under air (non-submerged) control plants, as indicated by the dashed line. Significance (*P* ≤0.05) and odds ratio indicate the results of a Fisher exact test over 5, 6, and 7 d of submergence. b) Fresh weight of old (O) and young (Y) leaves 1 d after the indicated submergence durations (in days, “d”) and 1 d of recovery. n = 10 leaves, mean ± SEM is shownSignificance of the 2-way ANOVA effects (*P* ≤0.05) are given in top corner. Here “t” is time, “a” the leaf-age. Letters above bars indicate differences from Tukey-HSD test (*P* < 0.05). c) The response to recovery of genes considered ABA induced. Genes that were among the top 250 induced genes 6 h after, and top 250 genes 24 h after 24 ABA treatment in leaf tissue (Cao et al. 2017) were considered ABA induced (441 genes). Significance values above boxplots concern, for the corresponding time point, the enrichment of ABA-induced genes among recovery induced genes compared with non-recovery induced genes (Fisher's exact test). Timepoints shown (1, 3, 6, 24 h) are hours post-submergence recovery. Boxplot drawing rules were: center line, median; box limits, upper and lower quartiles; whiskers, 1.5× interquartile range or most extreme point if less than 1.5× interquartile range; points, outliers. NS, not significant (*P* > 0.05). d) Heat map showing the expression of ABA biosynthesis and degradation genes at various timepoints following de-submergence (0, 1, 3, 6, and 24 h). e) Stomatal aperture of old and young leaves before and after 4 d of submergence followed by 3 h of recovery. n = 166 to 192 stomata per sample. f) Stomatal length and stomatal density on the adaxial side (n = 18 to 25 images per leaf type) of old and young leaves. g) Relative difference in stomatal conductivity between experimental groups. Here the relative conductivity is based exclusively on water loss via stomates. This amounted to the combined effect of the density of stomates, the area and estimated depth of each pore (Franks and Farquhar 2001). Pore area was estimated by an ellipse calculated from the stomatal pore width and length, depth was based on the guard cell width of closed stomates. Calculations are based on data presented in E and F. h) Stomatal aperture of old and young leaves before and after 4 d of submergence after treatment with 0 uM (mock), 50 uM, or 100 uM ABA (for 3 h). Per sample, between 350 to 700 stomata were measured from 3 to 5 leaves per group. For E-H the data are mean ± SEM (n = 5). Significance of the 2-way ANOVA effects are given in top corner of the graphs. Here “trea” is treatment effect, “non-sub” the control non-submerged treatment, and sub + rec as submergence followed 3 h of recovery. Age is the effect of either a young or old leaf. ABA is the effect of mock (0 uM) or ABA treatment (50 or 100 uM). Tre*age and subm*ABA indicate the corresponding interaction effects. Letters indicate differences between individual bars (*P* < 0.05, Tukey HSD).

During recovery, older leaves visibly dehydrated faster than young leaves (Fig. 1a). This trend was confirmed from fresh weight and water content measurements of old and young leaves recovering from different submergence durations (Fig. 3a, 3b). These 2 parameters of leaf dehydration thus confirmed more frequent dehydration in old leaves than in young leaves (*P* < 0.05, Fisher exact test). There was a positive correlation between dehydration and submergence duration. This was likely due to the inability of submergence damaged leaves to recover post-submergence. Consistent with this age-dependent leaf dehydration during the post-submergence phase, several genes associated with this process were regulated in an age-dependent manner (Fig. 2e). Notably, Cluster K6 with sustained upregulation in old leaves and transient induction young leaves (Fig. 2e) was enriched for genes associated with the GO category “response to ABA” (GO:0009737). To further evaluate this trend in correlation with differential leaf dehydration during recovery, we assessed the expression of 250 ABA responsive genes (Cao et al. 2017) during recovery in our dataset (Fig. 3c). Counterintuitively, we found an inverse correlation between ABA transcriptional response and dehydration. While old leaves showed a consistent ABA response across all recovery time points, the corresponding response in young leaves was transient. An assessment of the expression of ABA metabolic genes during recovery suggested no major leaf age–dependent differences in ABA metabolism during recovery (Fig. 3d). ABA is an important regulator of post-submergence recovery, primarily through control of stomatal regulation (Yeung et al. 2018). Accordingly, in an ABA biosynthesis mutant (*aba3-1*), the inability to restrict stomatal opening during recovery correlated with increased water loss and leaf dehydration compared with wild-type controls (Fig. S10). Guard cell–specific complementation of the *aba3-1* mutation (referred to as *MYB60:ABA3*) (Bauer et al. 2013) resulted in a rescue of the water loss phenotype observed in *aba3-1* mutants, with *MYB60:ABA3* dehydrating similar to wild-type controls following a 5-d submergence period (Fig. S10). These results suggest that stomatal ABA is important for reducing water loss in plants recovering from submergence. However, since degradation of the cuticle during submergence has also been associated with post-submergence water loss (Xie et al. 2020), we investigated whether submergence affected the permeability of the leaf cuticle in an age-dependent manner. Using toluidine blue uptake as a proxy (Tanaka et al. 2004), we observed a similar increase in cuticular permeability in both old and young leaves (Fig. S11).

Next we probed the possibility that faster dehydration of old leaves might be explained by a larger stomatal aperture or higher stomatal density. An assessment of stomatal traits for young and old leaves revealed that while stomatal aperture changes were similar for old and young leaves after 3 h of recovery (Fig. 3e), the latter had smaller stomatal size but higher stomatal density (Fig. 3f). Stomatal conductance is the net effect of pore area, depth, and density (Franks and Farquhar 2001). The combination of density, pore size, and aperture revealed that old leaves have limited capacity to decrease stomatal conductance after submergence, while young leaves do. However, estimates of their relative stomatal conductance suggest that young leaves, while more responsive, have a similar conductance during the recovery phase (Fig. 3g).

We also tested whether dehydration differences between old and young leaves could be attributed to differences in ABA sensitivity. We found that ABA sensitivity of old leaves was significantly reduced during the post-submergence phase (Fig. 3h). Whereas a submergence treatment did not affect the closure of guard cells in response to ABA in young leaves, it reduced the effect of ABA on the stomatal aperture of old leaves.

Thus, observed differences in water loss between old and young leaves could not be ascribed to either stomatal or cuticular conductance. This would imply that perhaps other factors such as mesophyll conductance, linked to traits such as leaf hydraulics, tortuosity, and aquaporins (Ferrio et al. 2012; Hommel et al. 2014; Diao et al. 2025), could explain greater water loss from old leaves, with the decline in ABA sensitivity further aggravating dehydration.

### ER stress signaling contributes to recovery of young leaves

**Figure 4:**
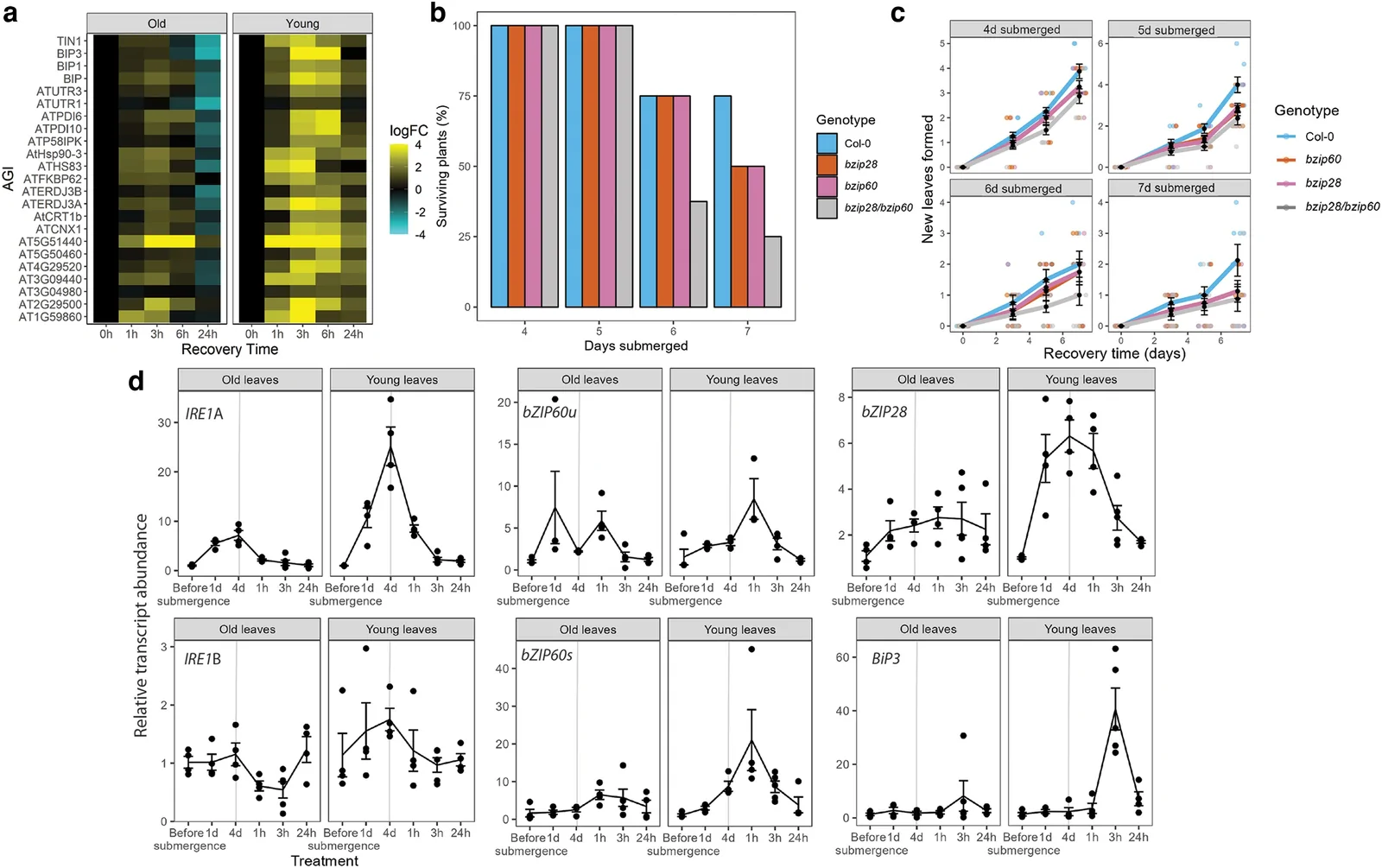
ER stress signaling contributes to post-submergence recovery in young tissues. a) Expression of transcripts in cluster 11 (Fig. 2e, Figure S8) associated with protein folding (GO:0006457) in young and old leaves during recovery, plotted relative to respective expression at various recovery durations following 4 d of submergence. b) Survival of Col-0, *bzip28*, *bzip60*, and *bzip28/60* plants after different submergence durations. Plants were submerged at the 10-leaf stage, desubmerged at the indicated submergence duration and allowed to recover under control growth conditions for 7 d. At this point survival was scored, with plants being scored as dead or alive based on the absence or presence of visible green tissues. At each time point survival was scored for 8 plants. c) New leaf formation of Col-0, *bzip28*, *bzip60*, and *bzip28/60* during recovery from different submergence durations. A significant genotype effect was observed during recovery, as determined by a 2-way ANOVA for recovery time* genotype (*P* < 0.05; error bar = SE). Per time-point, new leaf formation was scored for n = 8 plants. d) Transcript abundance of ER stress signaling pathway genes on old and young leaves during submergence (1 d, 4 d) and recovery following 4 d of submergence (1 h, 3 h, 24 h). Gray vertical line indicates end of submergence period and start of recovery. Mean is average of 3 biological replicates (n = 3; error = SE) with each consisting of 5 young or 5 old leaves from a single plant.

At the submergence durations used here, old leaves dehydrate, are unable to recover, and die. In contrast, younger shoot tissues resume growth following de-submergence. Among the biological processes identified as differentially regulated in young leaves were genes associated with ER stress and protein folding (Fig. 2e, Fig. 4a). We hypothesized that ER stress sensing and mitigation via activation of the unfolded protein response (UPR) during and after submergence contribute to post-submergence recovery in younger tissues.

The ER is the primary site of oxidative protein folding, achieved via a disulfide relay system comprising PROTEIN DISULFIDE ISOMERASE (PDI) and ER OXIDOREDUCTIN (ERO) enzymes. As molecular oxygen is the terminal electron acceptor for EROs, disulfide bond formation is sensitive to hypoxia (Ugalde et al. 2022). ER stress is triggered by stresses such as submergence and the concomitant reduction in oxygen availability (Gayral et al. 2017; Zhou et al. 2021; Gibbs et al. 2024), leading to an accumulation of misfolded or unfolded proteins in the ER. This elicits the UPR, which serves to restore homeostasis. Two classes of ER stress sensors are known in plants: membrane-associated basic leucine zipper (bZIP) TFs and RNA splicing factors, specifically INOSITOL-REQUIRING ENZYME TYPE 1 (IRE1). We tested the physiological significance of the observed ER-stress marker inductions using single and double mutant combinations for *bZIP60* and *bZIP28*, each mediating the 2 UPR activating pathways (Ko and Brandizzi 2022). bZIP28 is an ER stress sensor that directly mediates nuclear activation of stress-responsive genes. bZIP60 operates downstream of IRE1, activating the UPR following a splicing event that permits its translocation to the nucleus. We observed no developmental phenotypes associated with these mutations. However, there was a clear negative effect of submergence correlated with stress duration (Fig. 4b, Fig. 4c). Consistently, plant survival of the double mutant was reduced by more than 50% when plants were submerged for more than 6 d (Fig. 4b). Despite these phenotypes, all genotypes recovered and were able to produce new leaves (Fig. 4c). Nevertheless, this capacity for shoot regeneration was significantly diminished in *bzip28/bzip60* especially after long-term submergence (Fig. 4c). These results indicated that having 2 functional branches of the UPR is required for shoot regeneration and plant survival after de-submergence. Notably, the degree of senescence was not affected since there were no significant differences in chlorophyll content between the genotypes at any time point (Fig. S12).

To test the involvement of ER stress responses at the molecular level, we measured levels of ER stress markers in old and young shoot tissues by qPCR (Fig. 4d). Time-course analysis showed a more prevalent accumulation in younger leaves than in older tissues. Moreover, rapid accumulation of ER stress sensors *IRE1a*, *IRE1b*, and *bZIP28* was evident within 1d of submergence and maximal by the end of treatments (4 d). Levels of *bZIP60*u, a splicing target of IRE1 were greatest 1 h after de-submergence, as were the levels of the spliced version (*bZIP60*s). Lastly, *BINDING IMMUNOGLOBIN PROTEIN 3* (*BiP3*), a UPR gene induced by bZIP28 and bZIP60 was distinctly expressed only in the recovery period, 3 h after de-submergence. Similarly, mRNAs for other UPR genes were distinctly enhanced only after de-submergence (Fig. S13). This indicated that mRNAs for ER stress sensing and UPR accumulate at distinct phases of submergence. ER stress signals initiate during submergence, while the UPR is induced only during the recovery phase in young shoot tissues.

### The activation of ER stress signaling in young leaves is specific to the recovery phase

**Figure 5:**
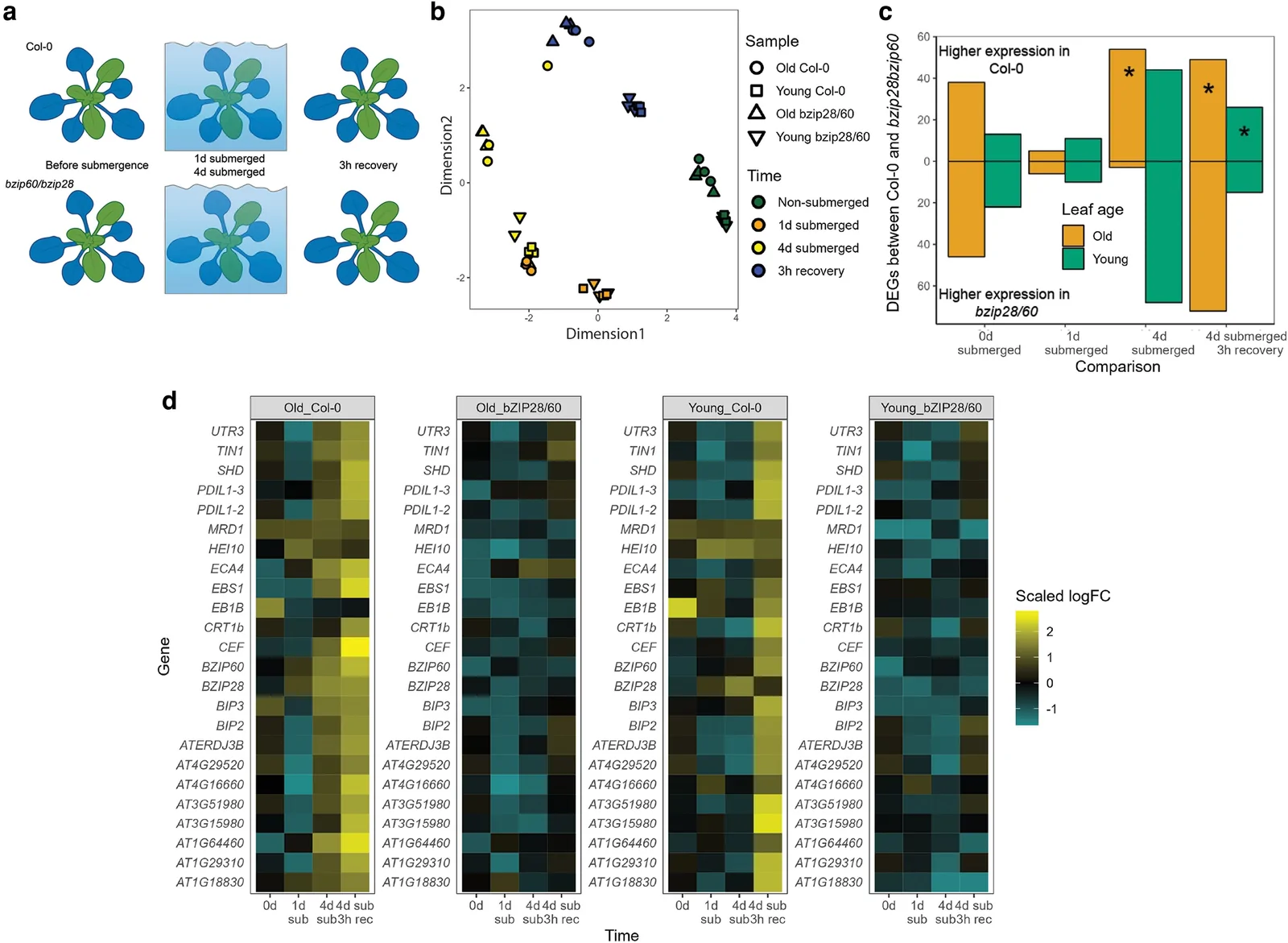
The role of ER stress signaling in transcriptional responses to submergence and recovery. a) Schematic of the harvested materials, young tissues are highlighted in green, old tissues are highlighted in blue. b) MDS plot of the old and young transcriptomes of Col-0 and *bzip28/60* before, during, and after submergence. c) The number of genes differentially expressed between Col-0 and *bzip28/60* in old and young leaves at each timepoint. Columns highlighted with an asterisk are significantly (Fisher exact test; *P* < 0.001; sample size for testing enrichment 27,430 DEGs) enriched for genes associated with the GO term “response to endoplasmic reticulum stress” (GO:0034976). d) Genes of which the expression pattern is significantly (*P* < 0.05) correlated with that of the ER stress marker gene *BIP3*, as determined by Pearson correlation coefficient. These contain multiple genes previously identified as being involved in the ER stress response, like *BIP2*, *UTR3*, *ATERDJ3B*, and *CTR1b*.

To further dissect the contributions of ER stress signals to the submergence response of Arabidopsis, we quantified whole genome and proteome effects. We hypothesized that transcriptome and proteome reconfiguration during submergence and recovery involves a combination of ER-dependent and -independent signals that are distinct in old and younger leaves. To determine the onset of responses that require ER stress signaling, we harvested old and young halves of Col-0 and *bzip28/60* rosettes before, during, and after submergence (Fig. 5a). Differential responses between each treatment and tissue type were separated over time by MDS, whereas the genotype-dependent responses were smaller (Fig. 5b; Table S2). Genotype-dependent responses were determined at each tissue and timepoint, and genes with an FDR-corrected *P* < 0.05 and an absolute fold change between the 2 genotypes were deemed as significantly different (Fig. 5c). Among the genes different between Col-0 and *bzip28/60*, genes in the GO category “response to endoplasmic reticulum stress” (GO:0034976) were enriched in the set with a higher expression in Col-0 old leaves after 4 d of submergence and after 3 h of recovery in both old and young leaves. This shows that the response to ER stress is gradually activated in old leaves during submergence and post-submergence recovery, whereas the response is only induced in young leaves during the post-submergence phase. Consistent with this, among genes that are significantly coexpressed with the ER stress marker gene *BiP3* are several that are known to play a role in ER stress signaling (Fig. 5d; Figs. S13 and S14). These all follow the pattern of a gradual induction during submergence that continues during recovery in old leaves and a recovery-specific induction in young leaves.

### Impact of the UPR on proteome responses to submergence-recovery stress

**Figure 6:**
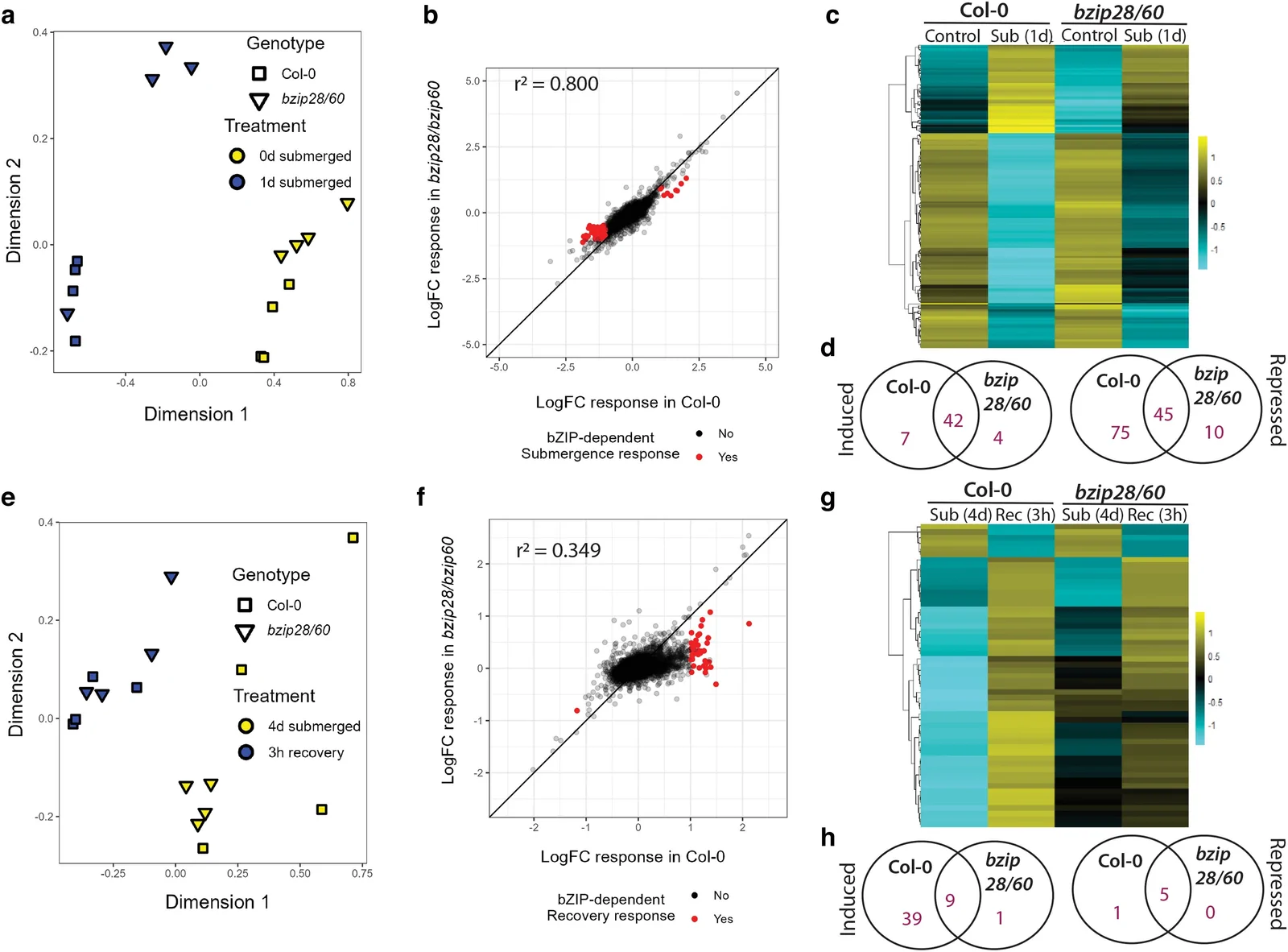
The role of ER stress signaling in submergence and post-submergence proteostasis. a) MDS plot of the proteomes of Col-0 and *bzip28/60* before and after 1 d of submergence; proteins with at least 2 unique peptides are shown. b) The response of proteins (4,767) to 1 day of submergence in Col-0 and *bzip28/60*. Proteins that show a response in Col-0 but not in *bzip28/60* are highlighted in red. Pearson r^2^ of the correlation in protein LogFC in both genotypes is shown in the top-left corner. c) Heatmap (log FC) of scaled protein abundance for 183 proteins with at least 2-fold changes (*P* < 0.05) in 1 comparison of Col-0 and *bzip28/60* before and after 1 d of submergence. d) Overlap between proteins induced and repressed in both genotypes after 1 d of submergence. e) MDS plot of the proteomes of Col-0 and *bzip28/60* after 4 d of submergence and 3 h of recovery. f) The response of proteins (4,767) to 3 h of post-submergence recovery in Col-0 and *bzip28/60*. Proteins that show a response in Col-0 but not in bzip28/60 are highlighted in red. Pearson r^2^ of the correlation in protein LogFC in both genotypes is shown in the top-left corner. g) Heatmap (log FC) of scaled protein abundance for 55 proteins with at least 2-fold changes (*P* < 0.05) in 1 comparison between Col-0 and *bzip28/60* after 4-d submergence and 3 h of post-submergence recovery. h) Overlap between proteins induced and repressed in both genotypes after 3 h of post-submergence recovery.

After establishing the consequences of the misregulation of ER stress signaling on the transcriptome, we sought to determine the global effects on the proteome. Considering that the demand for protein synthesis and oxidative protein folding in the ER is likely higher in young leaves and since the *bzip28/60* double mutant showed a slower rate of post-submergence leaf formation (Fig. 4d), we focused on the changes in the proteome of young leaves. We conducted 2 experiments using isobaric tandem mass tag (TMTpro) labeling to identify proteins with altered abundance in wild type and mutant in response to submergence recovery treatments. First, young leaves were harvested before and after 1 d of submergence. Dimensionality reduction via MDS showed a clear separation of samples by treatment and genotype (Fig. 6a). Then 3,608 proteins represented by a minimum of 2 peptides were identified and quantified (Table S3; Fig. S15a). The proteome after 1 d of submergence was largely similar in Col-0 and *bzip28/60* but with lower fold-changes in the mutant (Fig. 6b, 6c, 6d). Relatively few proteins increased in abundance, as defined by a fold change (FC) > 2 at *P* < 0.05; 49 in Col-0 and 46 in *bzip28/60* (Fig. 6c and 6d). Isobaric labeling datasets tend to underestimate fold-changes (Rauniyar and Yates 2014) and applying a more relaxed cutoff (FC > 1.5; *P* < 0.05) identified 118 and 89 proteins with increased abundance following submergence in wild type and mutant, respectively (Fig. S16a). In Col-0, these included proteins associated with sugar starvation responses: PEROXIDASE 21 (PER21), SYNAPTOTAGMIN-4 (STY46), METHYLCROTONYL-CoA CARBOXYLASE ALPHA CHAIN (MCCA), (ISOVALERYL-CoA-DEHYDROGEASE (IVD), KIN GAMMA SUBUNIT1 (KING1), TREHALOSE PHOSPHATE SYNTHASE8 (TPS8), UDP-D-GLUCOSE/UDP-D-GALACTOSE 4-EPIMERASE 1 (UGE1), BETA-XYLOSIDASE 1 (BXL1), BETA-GALACTOSIDASE 4 (BGAL4), PLASMA-MEMBRANE ASSOCIATED CATION-BINDING PROTEIN 1 (PCAP1), DORMANCY-ASSOCIATED PROTEIN-LIKE1 (DRM1), GLUTAMINE-DEPENDENT ASPARAGINE SYNTHASE 1 (ASN1) (Arias et al. 2014; Tarancón et al. 2017), and 2 ethylene biosynthetic enzymes, ACC OXIDASE (ACO)1 and ACO2. However, only 3 of the 49 core hypoxia proteins (ALCOHOL DEHYDROGENASE 1 (ADH1), PHYTOGLOBIN 1 (PGB1) and AT4G27450.1) were upregulated under submergence (Mustroph et al. 2009). A greater number of downregulated proteins was identified: 120 in Col-0 and 55 in *bzip28/60*; FC > 2; *P* < 0.05; 316 and 208 at FC > 1.5; *P* < 0.05. Col-0 proteins with decreased abundance upon submergence were enriched in KEGG pathways associated with photosynthesis, flavonoid biosynthesis, and N-glycan biosynthesis and protein processing in the ER. The latter included CALNEXIN (CNX)1 and CNX2, a SecY family protein and members of the oligosaccharyl transferase complex. Mitochondrial membrane proteins [including voltage dependent anion channels (VDACs), TRANSLOCASE OF THE OUTER MITOCHONDRIAL MEMBRANE (TOM40-1), TOM9-2, MITOCHONDRIAL PHOSPHATE CARRIER 3 (MPT3), and MITOCHONDRIAL DICARBOXYLATE/TRICARBOXYLATE TRANSPORTER (DTC)], and proteins associated with chromatin binding [including nucleosome assembly proteins, subunits of the facilitates chromatin transcription (FACT) complex and Imitation SWItch (ISWI) chromatin-remodeling complex ATPase] were also downregulated under submergence.

As genes involved in ER stress signaling were mostly induced during the post-submergence recovery phase (Fig. 5d), we also mapped the changes in the young leaf proteome during the first 3 h of post-submergence recovery after 4 d of submergence. The 4,767 proteins represented by a minimum of 2 peptides were identified and quantified (Table S4; Fig. S15b) and again, the samples grouped together based on both genotype and treatment (Fig. 6e). The proteome after 3 h recovery was largely similar in Col-0 and *bzip28/60* but with more deviation than the submergence response and lower fold-changes in the mutant (Fig. 6f, 6g, 6h; Fig. S16b). In the wild type, many of the protein categories that were downregulated under submergence increased in abundance after recovery, most notably mitochondrial membrane proteins (including VDACs), photosynthetic proteins, proteins associated with chromatin and splicing, and protein processing in the ER (Fig. S17). While the data did not bear the strong hallmark of the classical UPR response seen in the transcriptome, the proteome of the *bzip28/60* mutant had not recovered to the same extent as wild type after 3 h (Fig. 6f, Fig. 6g; Fig. S15b), consistent with a defect in ER protein processing. Relatively few proteins were downregulated upon recovery, although abundance of 3 photoreceptors: cryptochrome1, phytochrome A, and phototropin-2 was reduced in both mutant and wild type. Comparing scaled protein abundances revealed that VDAC1, 2, 3, and 4 are downregulated under submergence and accumulate during recovery, an effect that is less pronounced in the *bzip28/60* mutant compared with wild type (Fig. S18a). This prompted us to investigate the impact of VDAC function on submergence-recovery resilience. Rosettes at the 10-leaf stage were subjected to 6-d submergence and new leaf emergence was monitored over 3, 7, and 10 d of recovery (Fig. S18b-c). As already demonstrated for wild-type plants (Fig. 1; Rankenberg et al. 2024), *vdac1* and *vdac2* older leaves died and younger leaves and the meristem remained green. Monitoring new leaf formation over 10 d of recovery revealed that compared with wild-type Col-0 plants, *vdac1* was impaired in new leaf formation following submergence, whereas *vdac2* and *vdac3-1* mutants had a modest but statistically significantly increased rate of leaf formation (Fig. S18; Table S5). Taken together, these data suggest that VDACs might play distinct but overlapping roles in recovery from submergence.

## Discussion

Plant age, tissue, and cell-type identity can be important determinants of response and tolerance to environmental stresses. This is also the case for plant responses to flooding. Previous studies have explored the specific responses of plant organs or cell types to submergence and hypoxia, but few have addressed the effect of leaf age (Mustroph et al. 2006; Van Veen et al. 2016; Müller et al. 2021; Geldhof et al. 2024; Rankenberg et al. 2024). In this study we sought to characterize the molecular processes mediating the leaf age–dependent stress responses of *Arabidopsis thaliana* during submergence and recovery. The Arabidopsis rosette offers an excellent system to probe leaf age-dependent responses to submergence. Upon complete submergence, differential stress symptoms are clearly visible in terms of senescence and dehydration. During recovery, progression of symptoms occurs only in old leaves while young leaves recover growth and meristem activity is restored. Exploiting these differences, we sought to understand the molecular bases for this stress phenotype.

### Leaf age–dependent responses are most evident during recovery corresponding with considerable transcriptomic modulation

During complete submergence, in theory, the stress is imposed equally across the entire rosette. For example, initial changes related to submergence such as mechanical stress from the water column or the accumulation of ethylene are expected to be equal across the rosette. The assessment of the Arabidopsis rosette transcriptomes revealed substantial remodeling under stress. Surprisingly, young and old leaf responses showed a significant correlation during submergence (Fig. 2c). These core responses included several molecular processes typically activated in response to submergence stress, including response to ethylene and the downregulation of growth, photosynthesis, and translation. An exception was the response to hypoxia, which showed strong leaf age–dependent responses, although no clear trend was observed (Fig. S4). The similarity between the transcriptomes of a 4-d-submerged old leaf and a 6-d-submerged young leaf suggested that the difference between old and young leaves in their response to submergence was in part determined by the speed of their response. Submergence treatment thus induced “transcriptomic aging” of a leaf and old leaves were more sensitive to this induction than young leaves (Fig. 2d).

Leaf age–dependent transcriptome responses were most apparent during the recovery phase. The recovery period following submergence can be viewed as a sequential stress for plants. Transition to aerial light and oxygen conditions is associated with increased dehydration and oxidative stress (Yeung et al. 2018; Yeung et al. 2019). While these symptoms are most apparent and enhanced as the recovery period progresses, they are likely an outcome of a complex interaction of tissue physiology and stress responsiveness and a culmination of processes initiated during submergence and maintained or amplified during recovery (Yeung et al. 2019; Rankenberg et al. 2024). For example, superior recovery of young leaves (Fig. 2a) suggesting a higher tolerance of post-submergence stress is likely mediated by ROS protective mechanisms activated already during the submergence phase together with the further post-submergence induction of several pathways previously implicated in recovery. Quantitative differences in post-submergence dehydration and ROS accumulation contribute to differential submergence tolerance of the Arabidopsis accessions Bay-0 and Lp2-6 (Yeung et al. 2018). In the current study, the response to these stimuli was induced more strongly in young leaves than old leaves (Fig. 2e, Fig. S5). In addition, young leaves showed a stronger induction of genes associated with ER stress and the UPR. While old and young leaves both repressed processes associated with growth and photosynthesis during submergence, young leaves were able to restart these processes faster upon de-submergence. Many TFs were regulated in an age-dependent manner, although there were surprisingly few that exhibited opposite expression patterns between old and young leaves. This also applied to non-TF genes: differences between old and young leaves were mostly in the strength of the response rather than the direction of the response.

### Senescence and dehydration are initiated in older leaves

The ability to delay foliar senescence (the “stay-green” phenotype) is considered a valuable trait for sustained yield and biomass under control and stress conditions (Kamal et al. 2019), and, accordingly, the mechanisms underlying age-dependent leaf senescence have been extensively studied (Schippers 2015). Submergence presents a unique case where, despite systemic accumulation of ethylene (a well-known positive regulator of senescence), chlorophyll degradation still occurs in a leaf age–dependent manner. Mechanisms underlying this leaf age–dependent response to a leaf age–independent signal are not fully resolved (Rankenberg et al. 2021; Rankenberg et al. 2024). Consistent with the observed phenotype, processes related to senescence were induced faster in old leaves, but they were not enriched among genes with higher expression in old leaves under control conditions (Fig. S5). This suggests that the earlier onset of submergence-induced senescence in old leaves was not merely due to baseline differences in development but caused by differences in the speed at which senescence was activated. Age-related differences in the onset of senescence are associated with age-related changes that control when a leaf is sensitive to senescence-inducing signals (Jibran et al. 2013; Schippers 2015; Kanojia et al. 2020). An important regulator of chlorophyll catabolism in Arabidopsis is the NAC TF ORE1 (Qiu et al. 2015; Rankenberg et al. 2024). Although *ORE1* expression was strongly induced in both old and young leaves during submergence, its expression declined during the post-submergence phase in young leaves only (Fig. S19). The gradual derepression of ORE1 by *miR164* with leaf age is a prime example of these age-related changes (Jin et al. 2009), but few other mechanisms have been identified. We recently showed that post-translational ORE1 regulation mediates differential leaf-aging during submergence in a process that does not involve *miR164* (Rankenberg et al. 2024).

In the post-submergence phase, accelerated senescence can be linked to increased dehydration. Plants recovering from submergence stress typically experience physiological drought, as their damaged root systems are unable to compensate for water lost via the leaves (Yeung et al. 2019; Liu and Zwiazek 2022). Consistent with the observed phenotype, older leaves of submerged plants had an unfavorable water status compared with young leaves and stronger enrichment of clusters related to dehydration (Fig. 3a). Flooding is known to induce molecular and physiological responses similar to drought in various species, including a strong upregulation of ABA in shoots during waterlogging and submergence recovery (Yeung et al. 2018; Zagorščak et al. 2025). However, the exact role of ABA during submergence recovery and the correlation with drought tolerance remains unclear. While our transcriptome data revealed a more persistent ABA response in old leaves during recovery, there were no indications for differential transcriptional regulation of ABA biosynthesis. Greater post-submergence dehydration in the flood-sensitive Arabidopsis accession Bay-0 correlated with higher ABA levels and ABA-mediated induction of the guard cell localized *SENESCENCE-ASSOCIATED GENE 113* (*SAG113*) (Yeung et al. 2018). SAG113 mediates greater stomatal opening and water loss in older leaves to accelerate senescence. The higher expression of *SAG113* in old leaves in our dataset pointed to a similar mechanism underlying the greater dehydration of older leaves during recovery (Fig. S19). However, rather than stomatal movement, density, or stomatal conductance, our data suggest that leaf age–dependent dehydration differences during recovery are correlated with submergence-mediated reduction of ABA sensitivity in old leaves.

### ER stress responses contribute to submergence recovery in young leaves

During adverse conditions, the proper folding of secretory proteins in the ER can be disrupted, leading to an overload of misfolded proteins in the ER lumen and causing ER stress. This condition is managed by initiating the UPR, which serves to maintain ER homeostasis by aiding removal of misfolded proteins and enhancing protein folding capacity. A swift response to ER stress is essential for restoring cellular homeostasis and stress recovery. If the response is inadequate it can culminate in programmed cell death. UPR signaling in plants is mediated by 2 routes: involving the ER membrane–associated TFs bZIP17 and bZIP28 and the ER membrane–localized RNA-splicing factors IRE1/2 (Ko and Brandizzi 2024). Considering the oxygen dependency of protein folding in the ER (Fuchs et al. 2022), it is not surprising that flooded tissues experience ER stress. The induction of ER stress-associated transcripts observed here was consistent with previous studies showing the importance of the UPR for hypoxia and flooding (Zhou et al. 2021; Ugalde et al. 2022). However, our data suggest this has an underlying age dependency.

Our observation of a stronger ER stress signature in young leaves during recovery could suggest more severe ER stress (perhaps related to higher protein synthesis rates compared with old leaves) or a greater capacity to respond to ER stress. Assessment of mutants disabled in the 2 branches of the UPR signaling pathway revealed an impact on survival following long-term submergence characterized by a difference in new leaf formation, but not in senescence (which occurs in old leaves). This suggests that while flooding likely induced accumulation of misfolded proteins leading to ER stress in both old and young leaves, young tissues demonstrate ER stress recovery and adaptive strategies to permit continuation of growth, as reflected in resumption of new leaf formation. This was also supported by a stronger induction of upstream regulators (*IRE1a*, *bZIP60s*, *bZIP28*) and their downstream targets (eg *BiP3*) during recovery in young leaves. An assessment of the impact of ER stress on the proteome of young leaves revealed that protein processing in the ER is down regulated upon submergence in agreement with the oxygen-dependency of ER protein folding. However, initiation of UPR pathways as plant tissues transition to recovery conditions is essential as evidenced by a slower recovery in *bzip28/60* because the UPR is impaired. Our data also indicate that while ER stress is likely sensed during submergence, UPR activation occurs during recovery. Although not investigated here, this could be associated with the generation of signals such as ROS during this phase (Yeung et al. 2018; Gibbs et al. 2024; Khan et al. 2024; Yu et al. 2024).

The proteome comparisons between wild type and UPR mutants also pointed to a role for mitochondrial proteins during submergence recovery. Mitochondrial function is dramatically modulated during submergence recovery. Reduced oxygen availability under submergence inhibits the mitochondrial electron transport chain and subsequently, the restored respiratory capacity upon reoxygenation leads to a burst of ROS production (Chang et al. 2012; Sasidharan et al. 2018; Khan et al. 2024). Among the mitochondrial proteins whose abundance was affected both by the stress and the disruption of ER stress signaling were the VDACs. VDACs are the most abundant proteins of the mitochondrial outer membrane (Fuchs et al. 2020) and have overlapping and distinct functions in mitochondria (Tateda et al. 2011; Robert et al. 2012; Hemono et al. 2020); not only are they involved in exchange of metabolites and ions between the cytosol and the intermembrane space, but they also play roles in tRNA import and oxidative stress responses (Salinas et al. 2006; Hemono et al. 2020; Kanwar et al. 2022). The Arabidopsis genome encodes 6 VDAC genes, of which only 4 are expressed (Tateda et al. 2011; Robert et al. 2012). Assessment of the role of the VDACs revealed a potential role for VDAC1, the most abundant VDAC in the resumption of new leaf formation following desubmergence. Loss of VDAC3 function has been associated with oxidative stress tolerance (Kanwar et al. 2022), which may explain why *vdac3-1* and *vdac3-2* did not exhibit sensitivity to submergence stress and indeed perform better during recovery. Interestingly, a recent study identified VDAC1, VDAC2, and VDAC3 as mitophagy receptors, with corresponding mutants displaying mitophagy-associated phenotypes and being unable to clear damaged mitochondria. While these preliminary results point toward an involvement of the VDACs in submergence recovery, further investigation is needed to clarify their exact roles. Overall, our results suggest that the preferential activation of UPR related pathways downstream of bZIP17/28 and IRE1 are essential contributors to the superior recovery of young leaves.

### Spatiotemporal regulation of flood tolerance

Our work provides a comprehensive description of leaf-age dependent molecular responses during submergence, identifying mechanisms underlying age-specific leaf resilience and underscores the importance of the recovery phase. Plant resilience to environmental stresses is a complex interplay between developmental state and external changes. Additional complexity is conferred by the dynamic developmental progression and communication between different organs. For a leaf, such developmental changes might be linked to distinct physical and biochemical properties like leaf shape or antioxidant concentrations impacting stress tolerance. These are harder to change from a breeding perspective as this might impact plant performance also under non-stressed conditions. From the perspective of improving stress tolerance, while general resilience mechanisms have been identified [eg SUBMERGENCE1A (SUB1A) rice (Xu et al. 2006)], in some cases more targeted strategies might be desirable. Leaf age–specific mechanisms as described here provide important insights into the spatiotemporal finetuning of stress responses and also molecular pathways and regulatory components that might form important targets for spatiotemporal manipulation of crop traits.

## Materials and methods

### Plant material and stress treatments

Arabidopsis seeds of *bzip60/28* (Ruberti et al. 2018), *bzip28* (SALK_132285), and *bzip60* (SALK_050203) were donated by Dr. Federica Brandizzi from Michigan State University. Seeds of *vdac1* (SALK_034653), *vdac2* (SAIL_342_012), *vdac3-1* (SAIL_238_A01), *vdac3-2* (SALK_127899), and *vdac2 vdac 3-2* were donated by Prof Byung-Ho Kang and Dr Wenlong Ma from the Chinese University of Hong Kong (Ma et al. 2025). Seeds were stratified in 0.1% agar (about 1 mg of seeds per 1 mL of 0.1% agar) at 4 °C in the dark for 4 d. After stratification, 40 µL of seeds in 0.1% agar were sowed onto soil. Pots with seeds were germinated and grown in a growth chamber [20 °C, 200 μmol m^-2^ s^-1^ photosynthetically active radiation (PAR), a 9-h photoperiod from 8:00 to 17:00, and 70% relative humidity]. Pots were thinned when seedlings were at 2-leaf stage (with 2 true leaves and 2 cotyledons), so there was only 1 seedling per pot. Plants were grown to the 10-leaf stage before they were used for experiments. Flooding treatments were described previously (Yeung et al. 2018; Rankenberg et al. 2024). Plants were completely submerged in the dark at 20 °C for the indicated durations and left to recover for the indicated time in the original climate chamber.

### RNA-seq

For the Col-0 mRNAseq, leaf blades of leaf 3 (old leaf) and leaf 7 (young leaf) were harvested before submergence, after 2 and 4 d of submergence, and after 1, 3, 6, and 24 h of recovery (Fig. 1). Also, 8 to 16 leaf blades were pooled together per biological replicate. For the Col-0 and *bzip28/60* RNA-seq, Arabidopsis plants were harvested before submergence, after 1 and 4 d of submergence, and after 3 h of recovery in the light. Old (leaves 1 to 5) and young (leaves 6 to 10 and the meristem) halves of the rosettes were harvested separately. Two plants were pooled together per biological replicate. Three biological replicates were used per genotype, tissue type, and time point. For sampling times that were 24 h apart (t0_00, t2_00, t4_00, t6_00), harvesting always took place at the same time of day (10:00 h). Recovery time points harvests were at the indicated times (h) starting from 10:00 h.

Biological replicates were harvested independently. For both RNAseq experiments, RNA was isolated using the RNeasy Plant Mini kit (Qiagen), residual genomic DNA was digested using on-column DNase-I digestion (Ambion). RNA was quantified using a Nanodrop spectrophotometer, quality was analyzed by measuring A260/A280 and A230/A280 values on a Nanodrop and by running 1 µL of RNA on an agarose gel. Library preparation was performed commercially by Macrogen Korea using the TruSeq Stranded mRNA LT Sample Prep kit. The cDNA libraries were sequenced by paired-end Illumina sequencing on an Illumina Novaseq6000 platform, yielding approximately 30 million paired-end 150 bp reads per library. Illumina sequencing of cDNA libraries from mRNA isolated from harvested samples resulted in between 30 million and 47 million high-quality paired end reads per library (Fig. S20 and S21). These reads were then trimmed of adapter sequences and aligned to the Araport11 transcriptome yielding high mapping percentages (Fig. S20 and S21). The mapping percentage was notably lower for Col-0 young leaves harvested after 6 d of submergence. A brief analysis of the origin of reads that did not map to Arabidopsis using BLAST revealed that they originated from multiple oomycetes, some viruses, as well as some unidentified sources. This suggests that the harvested Arabidopsis plants were not infected by 1 specific virus, as is sometimes observed in RNAseq datasets (Verhoeven et al. 2023). We did not observe any obvious symptoms of pathogen infection or disease on the submergence treated plants.

### Read alignment and DEG calculation

FASTQ files were cleaned up using cutadapt (Martin 2011) to remove some residual sequencing primers, and the quality was assessed with FastQC (Babraham Bioinformatics). Cleaned up FASTQ files were aligned to the Arabidopsis Araport11 transcriptome using Kallisto (Bray et al. 2016) on the Utrecht Bioinformatics Cluster. Differential gene expression was calculated in R using Bioconductor packages edgeR and limma (Robinson et al. 2009; Ritchie et al. 2015). Only transcripts that were present at a minimum of 20 reads in 3 or more samples were included, and different transcript isoforms encoded from the same gene were merged. Genes were determined to be differentially expressed when they had an absolute log_2_ fold change above 1 and an FDR-corrected *P* < 0.05.

### Gene ontology and TF enrichment

Gene Ontology enrichment was calculated using the R package GOseq (Young et al. 2010). Enrichment of TF targets was done using the PlantRegMap database (Tian et al. 2020). Enrichment of the targets of each TF per cluster was tested by hypergeometric test, adjusted for multiple testing. For visualization, TF were grouped together based on their family, and the percentage of overrepresented TFs per family was shown to account for differences in TF family sizes.

### Water content

Rosettes were detached from the root system either before submergence or after the indicated submergence duration, rosettes cut off after submergence were gently blotted dry with a paper towel. Rosettes were kept on petri dishes at a relative humidity of approximately 55% and a temperature of 21 °C and were weighed on a Sartorius MX-5 scale at the indicated timepoints. After the last timepoint the plants were left at 80 °C for 2 d to dry out. Leaves were labeled as dehydrated when their water content (defined as (fresh weight—dry weight)/fresh weight * 100%) was lower than 90% of the mean water content of control leaves. A reduction of relative water content of 10% or more is typically associated with a stressful water deficit (Zhang and Bartels 2018). Another indication of the higher water loss of old leaves was their greater reduction in leaf fresh weight (Fig. 3b). Here, leaves with a fresh weight below 40% of the mean of non-submerged leaves were designated as stressed, as this was the lower boundary of the variation in fresh weight of untreated plants.

### Stomatal aperture

Adaxial sides of leaves were pressed into President dental paste at the indicated timepoints. Imprints were made of the adaxial side, as the abaxial side of the leaf was close to the wet soil and variation in humidity across the leaf microclimate can affect stomatal behavior (Pantin et al. 2012). After the imprints had hardened, leaves were removed and clear nail polish was applied to the imprints. Dried nail polish was mounted on microscope slides and was imaged on a Zeiss Axioskop II macroscope with a 100× magnification setting. Stomatal aperture was determined based on these pictures by measuring the ratio between the length and width of the stomatal pore using ImageJ (NIH).

### ABA treatment

ABA was applied to plants by dissolving ABA in ethanol and dissolving that in distilled water to a final concentration of 50 µM or 100 µM ABA, 0.1% Tween-20 was added to facilitate uptake of ABA. The mock solution contained 0.1% ethanol and 0.1% Tween-20 in distilled water. Plants recovering in the light from submergence in darkness were sprayed (200 µL per spray) with either the ABA solutions or the mock twice directly after de-submergence, once 30 min after de-submergence, and once 1.5 h after de-submergence. Stomatal aperture was determined using stomatal imprints 3 h after de-submergence.

### Toluidine blue staining

Old and young leaves were cut off at the indicated timepoints, and 2 pooled leaves of each were submerged in either 0.05% (w/v) Toluidine Blue solution or water for 2 min. Plants were washed 3 times in tap water to rinse off excess staining solution and were incubated for 4 h in 80% ethanol at room temperature in the dark to let all absorbed toluidine blue leach out of the leaf. Then 200 µL of each ethanol solution was measured for its absorbance at 626 nm in a Biotek HT plate reader. Leaves were dried for 48 h at 80 °C and were weighed to normalize absorbance at 626 nm by dry weight.

### Chlorophyll content

Chlorophyll extraction was performed in the dark. Each rosette was placed in a 2-mL tube and added with 1 mL DMSO. Samples were incubated at 65 °C shaking bath for 30 min, cooled down on ice for 1 min, and left at room temperature. Absorbances of 664, 647, and 750 nm were measured using a spectrophotometer plate reader (Synergy HT Multi-Detection Microplate Reader). Concentrations of chlorophyll a and chlorophyll b were calculated using the equations from (Porra et al. 1989) and normalized by dry weight. Tubes containing rosettes were dried at 80 °C for 1 wk. The dry weight was measured with a microbalance scale.

### Submergence phenotype scoring

New leaf formation and dead leaf counting were done following de-submergence at different time-points during recovery under control growth conditions. New leaves were counted by marking the youngest leaf following de-submergence and then monitoring the emergence of newer leaves during recovery. Leaves were described as dead when approximately more than half of the leaf lamina had desiccated (Fig. S22).

### q-PCR

Each sample includes 2.5 μL 2× SybrGreen Supermix, 0.25 μL of 10 μM forward primer, 0.25 μL of 10 μM reverse primer and 2 μL of 5 ng/μL cDNA. The qPCR was conducted on Applied Biosystems ViiA 7 Real-Time PCR System (Thermo Fisher Scientific). Forward and reverse primers of target genes we monitored are shown in Table S6. Relative mRNA abundance was calculated by the 2^−ΔΔCT^ method as presented by Livak and Schmittgen 2001.

### Proteomics

Arabidopsis plants Col-0 and *bzip28/60* were sampled before submergence, after 1 and 4 d of submergence, and after 3 h of recovery in the light. Young rosettes (leaves 6 to 10 and the meristem) were harvested. Three plants were pooled together per replicate and 4 replicates were harvested. Protein extraction, quantification, reduction, and alkalization were done as in Zhang et al. (2015). Protein precipitation was done by the methanol/chloroform method as in Zhang et al. (2018) and sequential trypsin digestion performed at a protease: protein ratio of from 1:100 to final 1:50 (w/w). Peptide concentration was determined using a Pierce Quantitative Colorimetric Peptide Assay kit (23,275, Thermo Scientific). Labeling was performed by mixing 100 µL of peptide (1 µg/µL) with 40 µL (∼0.25 mg) TMTpro stock in acetonitrile (TMTpro 16plex Mass Tag Isobaric Labeling Reagent Set Thermo Fisher Scientific) and then incubated for 1 h at room temperature.

This was followed by quenching with 8 µL of 5% hydroxylamine solution and incubation at room temperature for 15 min. Equal amounts of each prepared peptide were combined in a new tube. The solvent composition was adjusted to acetonitrile <5% and 1% trifluoracetic acid (HPLC grade 99.5% 044630.AE Alfa Aesar) for desalting with Sep-Pak vac tC18 cartridge [100 mg] (Waters).

#### High-pH first dimension reverse-phase fractionation

The following LC conditions were used for the fractionation of the TMT samples: desalted peptides were resuspended in 0.1 mL 20 mM ammonium formate (pH 10.0) + 4% (v/v) acetonitrile. Peptides were loaded onto an Acquity bridged ethyl hybrid C18 UPLC column (Waters; 2.1-mm i.d.×150 mm, 1.7-µm particle size), and profiled with a linear gradient of 5 to 60% acetonitrile + 20 mM ammonium formate (pH 10.0) over 60 min, at a flow-rate of 0.25 mL/min. Chromatographic performance was monitored by sampling eluate with a diode array detector (Acquity UPLC, Waters) scanning between wavelengths of 200 and 400 nm. Samples were collected in 1-min increments and reduced to dryness by vacuum centrifugation.

#### LC-MS/MS

Dried fractions from the high pH reverse-phase separations were resuspended in 0.1% formic acid and placed into a glass vial. Then 1 μL of each fraction was injected by the HPLC autosampler and separated by the LC method detailed below. Fractions were combined into pairs and were analyzed by LC-MS/MS.

LC-MS/MS experiments were performed using a Dionex Ultimate 3000 RSLC nanoUPLC (Thermo Fisher Scientific Inc, Waltham, MA, USA) system and a Orbitrap Lumos Tribrid mass spectrometer (Thermo Fisher Scientific Inc). Peptides were loaded onto a pre-column (Thermo Scientific PepMap 100 C18, 5 mm particle size, 100 Å pore size, 300 mm i.d.×5 mm length) from the Ultimate 3000 auto-sampler with 0.1% formic acid for 3 min at a flow rate of 10 μL/min. After this period, the column valve was switched to allow elution of peptides from the pre-column onto the analytical column. Separation of peptides was performed by C18 reverse-phase chromatography at a flow rate of 300 nL/min using a Thermo Scientific reverse-phase nano Easy-spray column (Thermo Scientific PepMap C18, 2-mm particle size, 100-Å pore size, 75-mm i.d.×50 cm length). Solvent A was water + 0.1% formic acid and solvent B was 80% acetonitrile, 20% water + 0.1% formic acid. The linear gradient employed was 2 to 40% B in 93 min. (Total LC run time was 120 min, including a high organic wash step and column re-equilibration).

The eluted peptides from the C18 column LC eluant were sprayed into the mass spectrometer by means of an Easy-Spray source (Thermo Fisher Scientific Inc.). All m/z values of eluting peptide ions were measured in an Orbitrap mass analyzer, set at a resolution of 120,000 and were scanned between m/z 380 to 1,500 Da. Data-dependent MS/MS scans (Top Speed) were employed to automatically isolate and fragment precursor ions by collision-induced dissociation [CID, normalized collision energy (NCE): 35%], which were analyzed in the linear ion trap. Singly charged ions and ions with unassigned charge states were excluded from being selected for MS/MS and a dynamic exclusion window of 70 s was employed. The top 10 most abundant fragment ions from each MS/MS event were then selected for a further stage of fragmentation by Synchronous Precursor Selection (SPS) MS3 (1) in the HCD high energy collision cell using HCD [High energy Collisional Dissociation, (NCE: 55%)]. The m/z values and relative abundances of each reporter ion and all fragments (mass range from 100 to 500 Da) in each MS3 step were measured in the Orbitrap analyzer, which was set at a resolution of 50,000. This was performed in cycles of 10 MS3 events before the Lumos instrument reverted to scanning the m/z ratios of the intact peptide ions and the cycle continued.

Raw data were searched against the Arabidopsis TAIR10; cRAP (version of the data) database using MASCOT version 2.7.0 (Matrix Science, London, UK) and PROTEOME DISCOVERERTM v. 2.5.0.400 as described previously employing Top 10 peaks filter node and percolator nodes and reporter ions quantifier with Trypsin enzyme specificity with a maximum of 1 missed cleavage. Carbamidomethylation (+57.021 Da) of cysteine and TMTpro isobaric labeling (+304.207) of lysine and N-termini were set as static modifications while methionine oxidation (+15.996) was considered dynamic. Mass tolerances were set to 10 ppm for MS and 0.8 Da for MS/MS. For quantification, TMTpro method was used, and integration tolerance was set to 20 ppm, Integration Method was set as centroid sum. Purity correction factor was set according the TMTpro product sheet. Each reporting ion was divided by the sum of total ions and normalized by medians of each sample. Log transformation ensured a homogeneity and normal distribution of the variances. Only proteins represented by 2 or more peptides were considered for further analysis. Heatmaps were generated by applying a z-score transformation using normalized log2-transformed abundance before performing the hierarchical clustering. Differential expression of proteins was determined using limma (Ritchie et al. 2015). Proteins were designated as differentially expressed when their FDR-adjusted *P* < 0.05 and their absolute log2 fold change was above 1.

### Accession numbers

The accession numbers (gene IDs) of the major genes mentioned in this study are provided in the Supplementary Tables.

## Supplementary Material

kiag317_Supplementary_Data

## Data Availability

mRNAseq data have been deposited at the European Nucleotide Archive under accession number PRJEB57289 (Col-0) and ArrayExpress accession E-MTAB-15812 (Col-0 vs *bzip* mutants). MS proteomics data have been deposited to the ProteomeXchange Consortium via the PRIDE partner repository (Perez-Riverol et al. 2022) with the dataset identifier PXD067068. A subset of the Col-0 mRNAseq results (PRJEB57289; 4d sub, 6 h recovery) were previously published in a comparative analysis with the *ore1-1* mutant (Rankenberg et al. 2024). All other datasets are original.
