# Supplementary material for: Distinct processes contributing to post-submergence recovery determine leaf age-specific flood resilience in Arabidopsis
Source: Plant Physiol. 2026 May 26;202(1):kiag317. doi: 10.1093/plphys/kiag317 (PMC13591405; doi:10.1093/plphys/kiag317)
Supplement: Supplementary file 1 [file kiag317_supplementary_data.zip › Rankenberg_Supplementary-POSTPROD_clean.pdf]

Supplementary figures:

Leaf-age specific flood resilience in *Arabidopsis thaliana* is determined by distinct processes contributing to post-submergence recovery

Tom Rankenberg[1], Maria Angelica Sanclemente[1,2,3], Hongtao Zhang[4] , Omar Orozco-Granados[1],Hendrika ACF Leeggangers[1], Muthanna Biddanda Devaiah[5], Janet R Chao[1], Hans van Veen[1,6] , Frederica L. Theodoulou[4], Rashmi Sasidharan[1]

[1] Plant Stress Resilience, Utrecht University, The Netherlands
[2] Agricultural Sciences, Clemson University, United States
[3] Vegetable Laboratory, US Department of Agriculture, United States
[4] Rothamsted Research, Harpenden, UK
[5] Groningen Institute for Evolutionary Life Sciences, The Netherlands
[6] Experimental and Computational Plant Development, The Netherlands

A

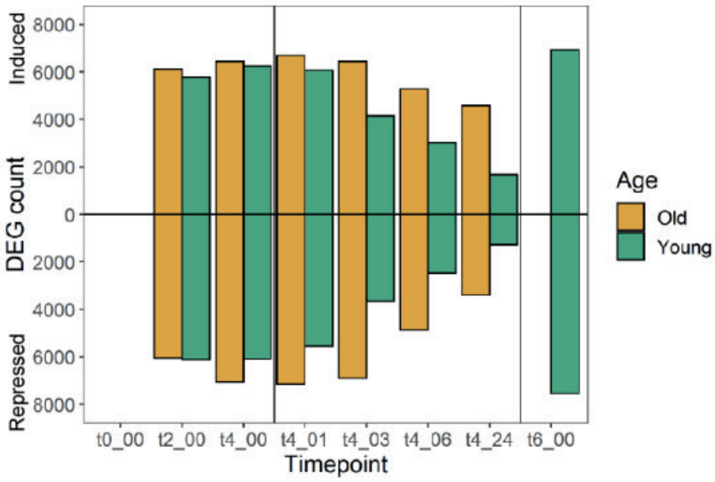

B

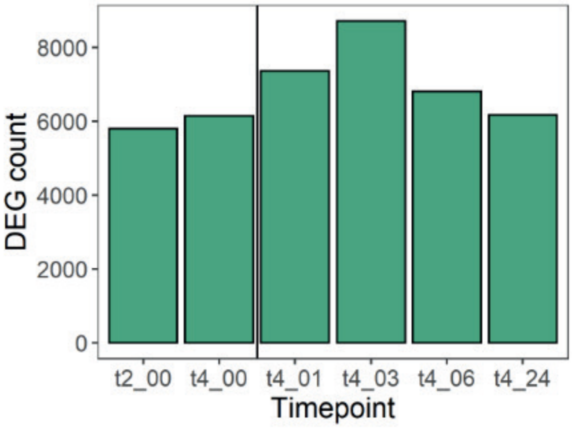

Supplementary figure S1: Differentially expressed gene (DEG) count for transcriptomics dataset.

A) DEG count for genes that responded significantly at the submergence or post-submergence timepoints compared to their non-submerged expression for old and young leaves separately. The first vertical line separates the submergence phase from the post- submergence phase. The second vertical line separates t6_00 from the samples recovering from four days of submergence.

B) DEG count for genes of which the difference in expression between old and young leaves changes significantly at each timepoint compared to non-submerged conditions. The vertical line indicates the separation between the submergence and post- submergence phases.

For both A and B, differential expression was calculated based on sample size of n=3 biological replicates (also see Methods).

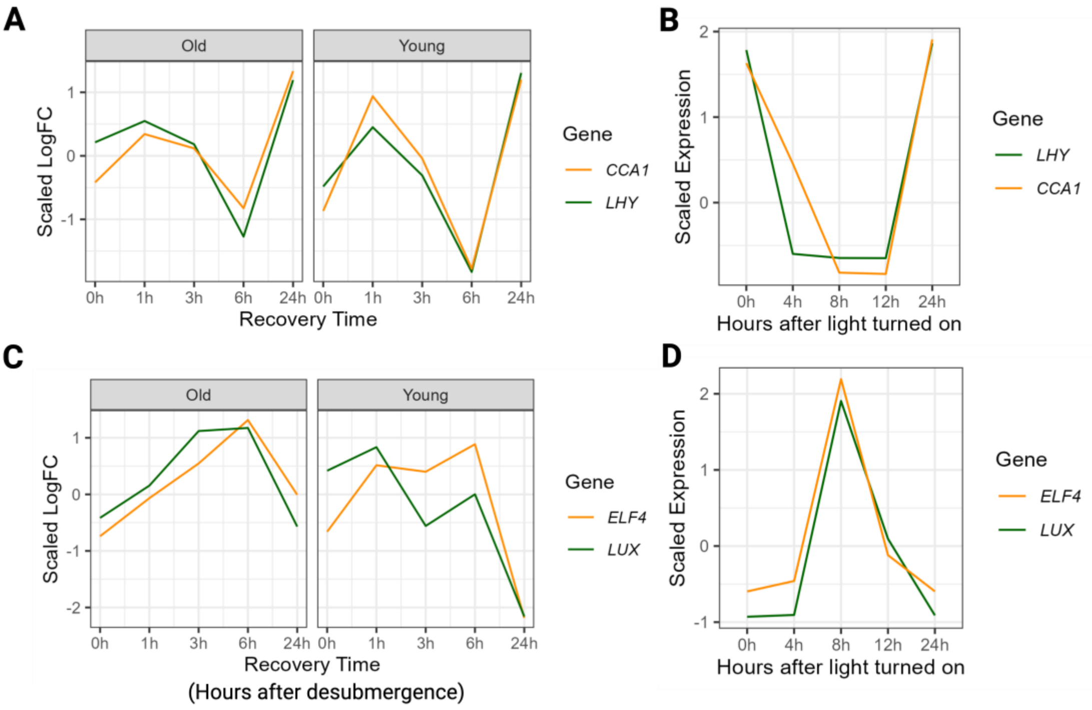

Supplementary figure S2: Assessment of circadian effects during submergence recovery. The transcript patterns of the circadian marker genes *CCA1* (AT2G46830), *LHY* (AT1G01060), *ELF4* (AT2G40080) and *LUX* (AT5G59570) in the current mRNAseq dataset (n= 3 biological replicates) of old and young Col-0 leaves (A, C) at the indicated recovery time points following 4 days of complete submergence (B, D). The transcript patterns of the same genes recorded in a previously published study from Michael et al. 2008 and showing the classical rhythmic cycling of circadian genes. Michael et al. 2008 data is based on Affymetrix ATH1 genechips in Arabidopsis Ler (short-day conditions) at indicated times following the start of the photoperiod.

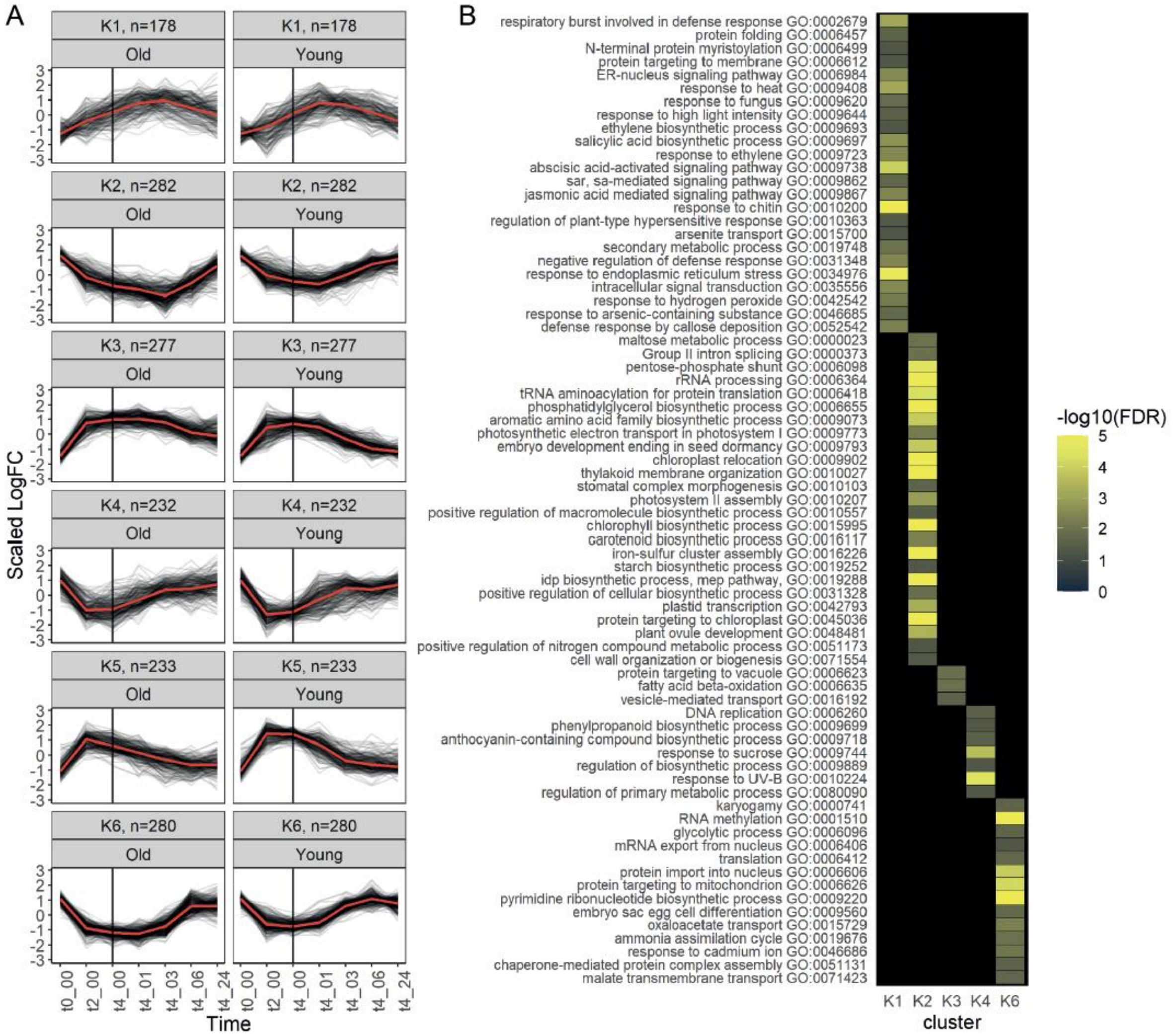

Supplementary figure S3: Age-independent transcriptomic responses to submergence

A) Fuzzy clustering of genes whose expression was different from non-submerged conditions in at least two samples, that never show an age dependent response. 1629 genes were divided over six clusters, yielding 1482 genes with a minimum cluster membership of 50%. Each black line represents a single gene, each red line represents the median expression in each cluster. The vertical black line indicates the separation between the submergence and the post-submergence phase.

B) Gene ontology (GO) enrichment of biological process terms in each of the six clusters shown in Figure 3A. All shown terms are significantly overrepresented in their respective cluster with an FDR-corrected p-value below 0.05.

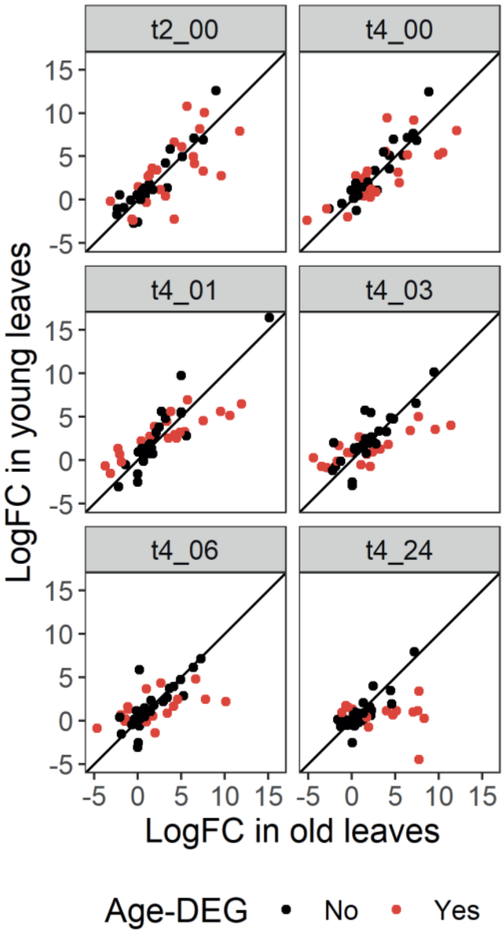

Supplementary figure S4: Core hypoxia genes in mRNAseq data

The response of the Arabidopsis core hypoxia gene set (Mustroph et al., 2009) (47 genes) to the indicated treatments. Genes with a significantly age-dependent response to the submergence or post submergence treatments are indicated in red. The diagonal line indicates where the response in old leaves (X-axis) is the same as that in young leaves (Y-axis).

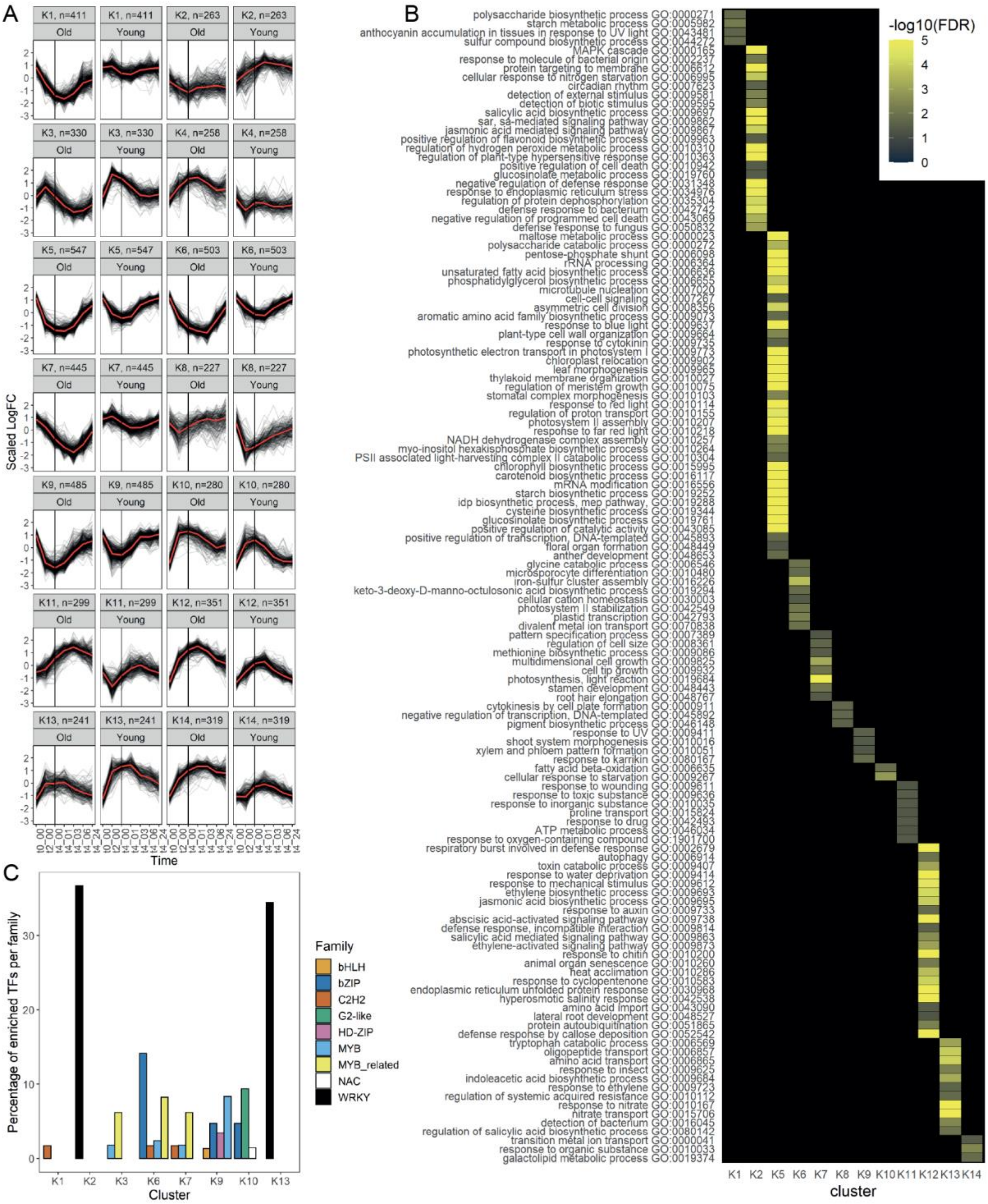

Supplementary figure S5: The age-dependent transcriptome response to the submergence and post submergence phase

A) Clustering of genes whose expression showed an age-dependent response at a minimum of four timepoints. 4977 genes were divided over 14 clusters, yielding 4959 genes with a minimum highest cluster membership of 20%. Each black line represents a single gene, each red line represents the median expression in each cluster. The vertical black line indicates the separation between the submergence and post-submergence phase.

B) Gene ontology enrichment of the biological process terms in each of the 14 clusters shown in Figure 4A. All shown terms were significantly overrepresented in their respective cluster with an FDR-corrected p-value below 0.05.

C) Enrichment of targets of transcription factors (TFs) in each cluster, grouped by TF family (Sample size for testing target enrichment 28643 genes). Enrichment was calculated by hypergeometric tests, corrected for multiple testing. Families with fewer than 2 TFs with overrepresented targets were omitted.

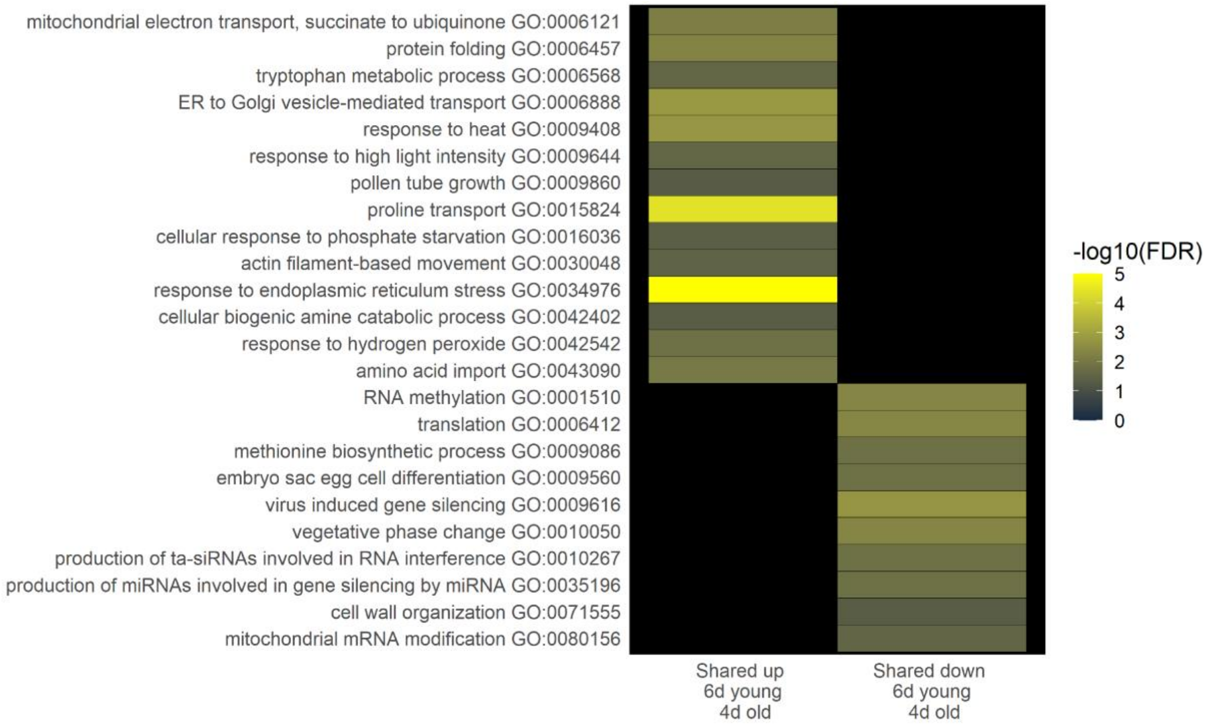

Supplementary figure S6: Significantly enriched biological process GO categories among genes that were up- or downregulated in old leaves after four days of submergence and in young leaves after six days of submergence. These were the last harvested submergence timepoints for old and young leaves. All shown terms were significantly overrepresented in their respective overlapping dataset with an FDR-corrected p-value below 0.05.

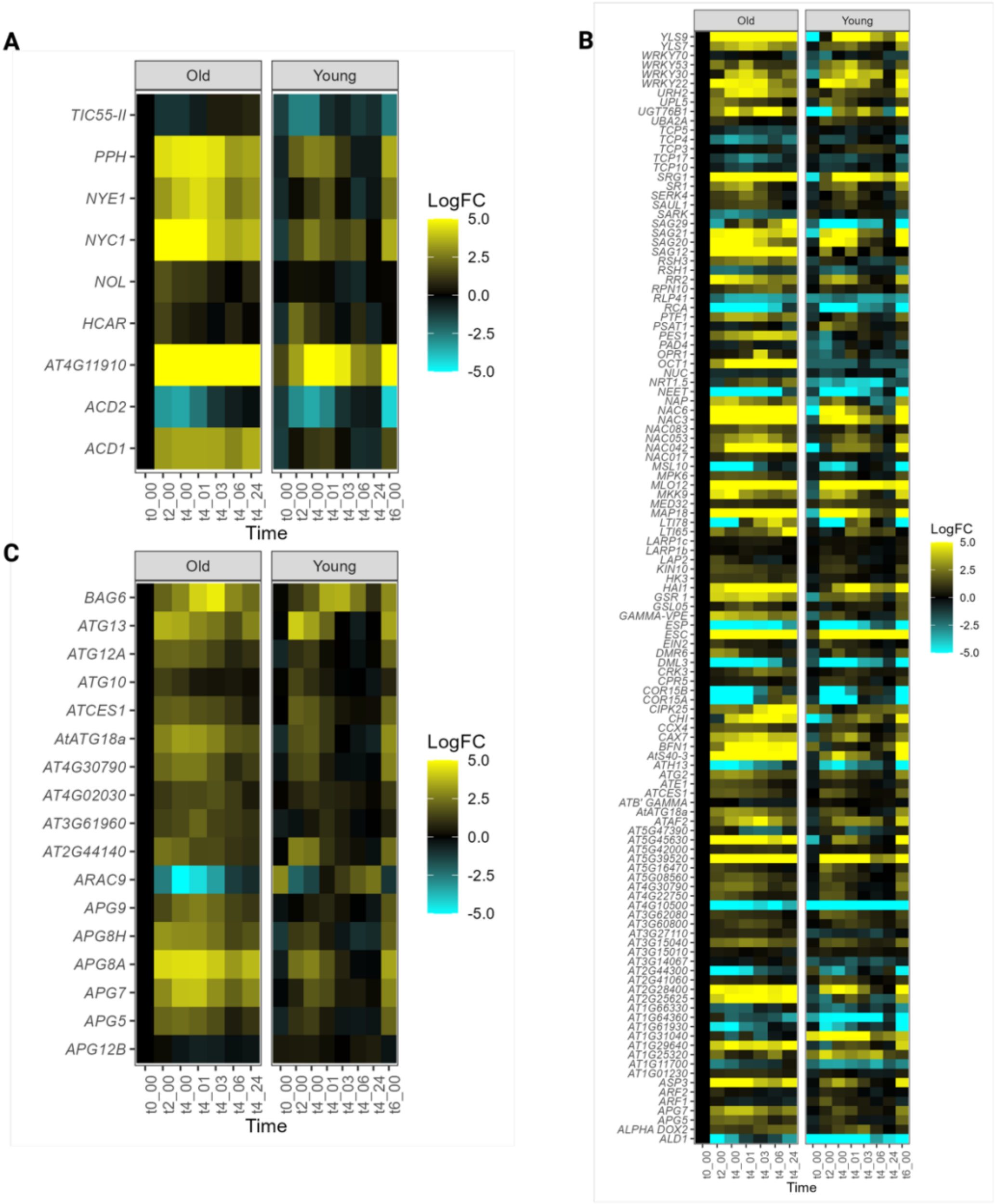

Supplementary figure S7: Age-dependent dynamics of leaf senescence and chlorophyll catabolism genes. Transcript abundance (log FC) of genes associated with (A) leaf senescence (GO:0010150), (B) chlorophyll catabolism (Kuai et al., 2016) and (C) autophagy (GO:0006914) in old and young leaves before (t0_00), during (t2_00, t4_00, t6_00) or after submergence (recovery, t4_01, t4_03, t4_06, t4_24).

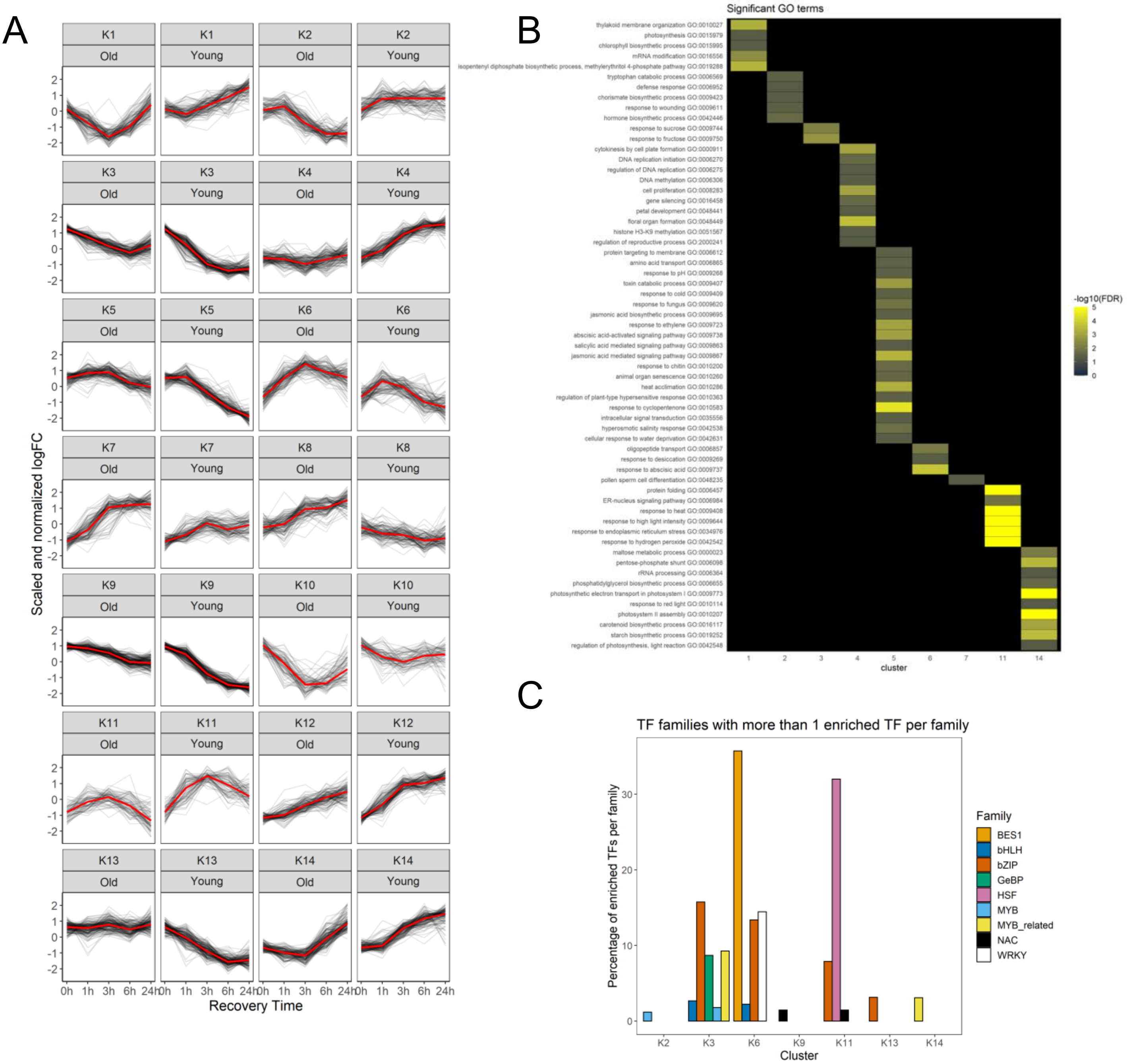
A
K1
Old
Young
K2
K3
K4
K5
K6
K7
K8
K9
K10
K11
K12
K13
K14
Scaled and normalized logFC
0h 1h 3h 6h 24h
Recovery Time
B
Significant GO terms
thylakoid membrane organization GO:0010027
photosynthesis GO:0015979
chlorophyll biosynthetic process GO:0015995
mRNA modification GO:0016556
isopentenyl diphosphate biosynthetic process, methylerythritol 4-phosphate pathway GO:0019288
tryptophan catabolic process GO:0006569
defense response GO:0006952
chorismate biosynthetic process GO:0009423
response to wounding GO:0009611
hormone biosynthetic process GO:0042446
response to sucrose GO:0009744
response to fructose GO:0009750
cytokinesis by cell plate formation GO:0000911
DNA replication initiation GO:0006270
regulation of DNA replication GO:0006275
DNA methylation GO:0006306
cell proliferation GO:0008283
gene silencing GO:0016458
petal development GO:0048441
floral organ formation GO:0048449
histone H3-K9 methylation GO:0051567
regulation of reproductive process GO:2000241
protein targeting to membrane GO:0006612
amino acid transport GO:0006865
response to pH GO:0009268
toxin catabolic process GO:0009407
response to cold GO:0009409
response to fungus GO:0009620
jasmonic acid biosynthetic process GO:0009695
response to ethylene GO:0009723
abscisic acid-activated signaling pathway GO:0009738
salicylic acid mediated signaling pathway GO:0009863
jasmonic acid mediated signaling pathway GO:0009867
response to chitin GO:0010200
animal organ senescence GO:0010260
heat acclimation GO:0010286
regulation of plant-type hypersensitive response GO:0010363
response to cyclopentenone GO:0010583
intracellular signal transduction GO:0035556
hyperosmotic salinity response GO:0042538
cellular response to water deprivation GO:0042631
oligopeptide transport GO:0006857
response to desiccation GO:0009269
response to abscisic acid GO:0009737
pollen sperm cell differentiation GO:0048235
protein folding GO:0006457
ER-nucleus signaling pathway GO:0006984
response to heat GO:0009408
response to high light intensity GO:0009644
response to endoplasmic reticulum stress GO:0034976
response to hydrogen peroxide GO:0042542
maltose metabolic process GO:0000023
pentose-phosphate shunt GO:0006098
rRNA processing GO:0006364
phosphatidylglycerol biosynthetic process GO:0006655
photosynthetic electron transport in photosystem I GO:0009773
response to red light GO:0010114
photosystem II assembly GO:0010207
carotenoid biosynthetic process GO:0016117
starch biosynthetic process GO:0019252
regulation of photosynthesis, light reaction GO:0042548
-log10(FDR)
cluster
C
TF families with more than 1 enriched TF per family
Percentage of enriched TFs per family
Cluster
Family
BES1
bHLH
bZIP
GeBP
HSF
MYB
MYB_related
NAC
WRKY

Supplementary figure S8: The age-dependent transcriptome response to post-submergence recovery

A) Clustering of genes whose expression shows an age-dependent response at a minimum of three recovery timepoints. 1848 genes were divided over 14 clusters, yielding 1845 genes with a minimum cluster membership of 20%. Each black line represents a single gene, each red line represents the median expression in each cluster.

B) Gene ontology (GO) enrichment of each of the 14 clusters shown in Figure 7A.

C) Enrichment of targets of transcription factors (TFs) in each cluster, grouped by TF family. Sample size for testing target enrichment is 28643 genes.

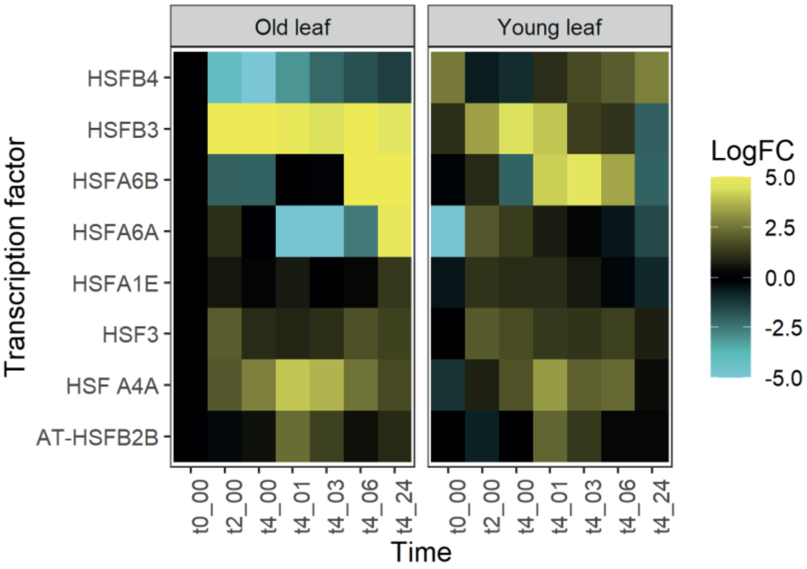

Supplementary figure S9: Expression of HSF transcription factors.

Expression of the eight transcription factors of the HSF family whose targets are enriched in cluster K11 (Supplementary figure S6). Expression was calculated relative to that of non-submerged old leaves and is shown for all the different sampling points (see Figure 1 and methods).

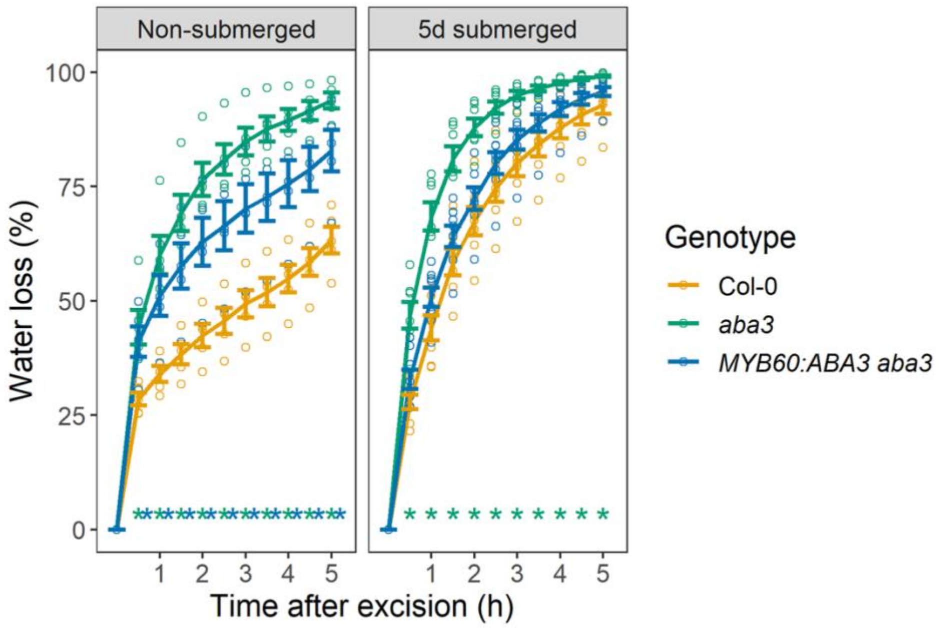

Supplementary figure S10: ABA biosynthesis contributes to limiting post-submergence dehydration. Relative water loss measured in wild-type (Col-0), an ABA biosynthesis mutant (*aba3-1*), and *MYB60:ABA3 aba3* plants (guard cell specific promoter mediated complementation of *ABA3* function in stomata) in the recovery period following 5d submergence. Rosettes were excised immediately following desubmergence and then weighed every 30 minutes, n=5-8 plants per genotype; (values shown are mean±SE; $P<0.05$). Relative water loss was calculated using the formula $1-((FW_{tX}-DW)/(FW_{t0}-DW))*100\%$, where FW_{tX} is the fresh weight at a given time point, FW_{t0} is the fresh weight immediately following excision of the rosette, and DW is the dry weight. Asterisks indicate significant differences from Col-0 at the indicated time point (One-way ANOVA with Dunnett's post-hoc test).

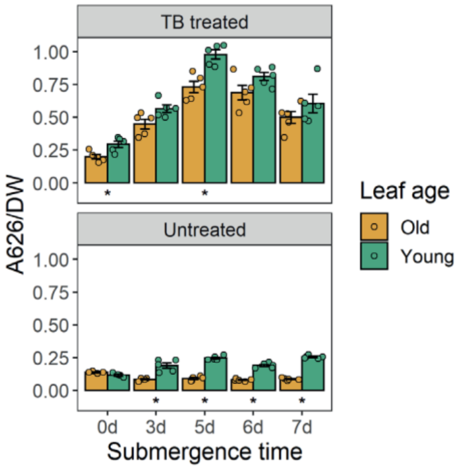

Supplementary figure S11: Cuticular permeability as measured by toluidine blue (TB) uptake of old and young leaves. Absorbance was measured at 626nm, values were normalized by leaf dry weight. Leaves not treated with TB were used to control for baseline differences in absorbance at 626nm. n=4-5 per group (values shown are mean±SE; $P<0.05$), each consisting of two old or young leaves from different plants pooled together. Asterisks indicate significant differences between old and young leaves (paired t-test).

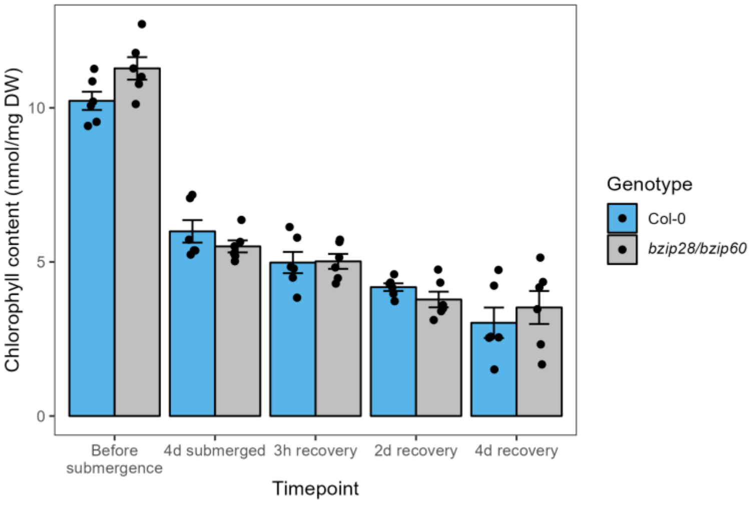

Supplementary figure S12: Senescence is not affected by disruption of the ER sensing pathway. Chlorophyll levels were measured for Col-0 and *bzip28/bzip60* double mutant plants submerged for 4d and then allowed to recover (3h, 2d, 4d). Data represents standard errors of the mean (SEM) where n=6. Two-way ANOVA with Tukey's multiple comparisons test was performed and found not to be significant for genotype comparisons $p<0.05$.

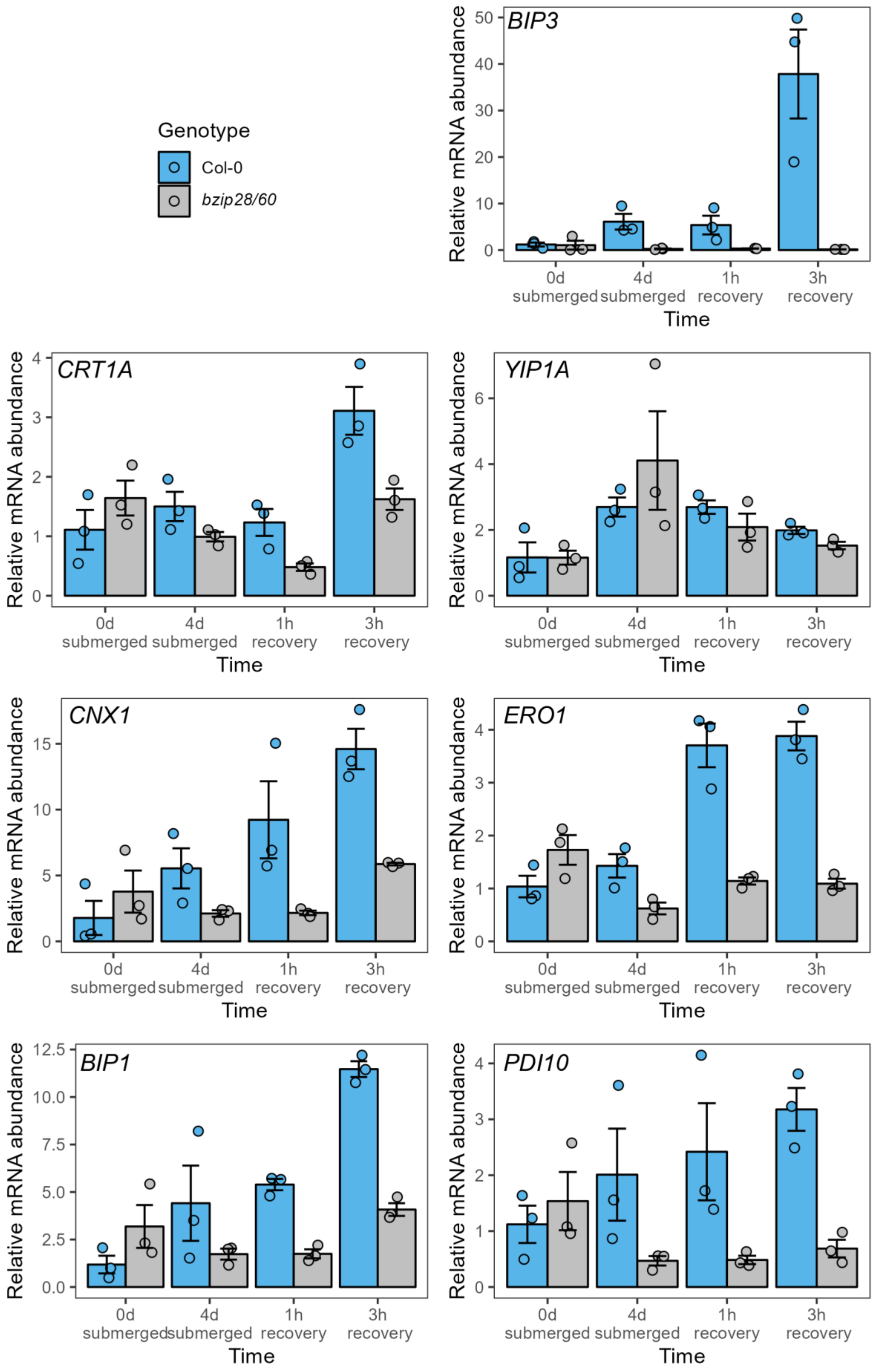
Genotype
Col-0
bzip28/60
BIP3
CRT1A
YIP1A
CNX1
ERO1
BIP1
PDI10
Relative mRNA abundance
0d submerged
4d submerged
1h recovery
3h recovery
Time

Supplementary figure S13: Unfolded Protein Response (UPR) genes are predominantly accumulated during the recovery phase. Plants were submerged for 4d and allowed to recover for three hours. Shoot tissue was collected from Col-0 and *bzip60/28* plants and mRNA levels were measured by qPCR using the *Actin2* gene as a normalization control. Quantified genes included *BIP1, BIP3* (*Binding protein 1 and 3*), *CNX1*(*Calnexin 1*), *CRT1A* (*Calreticulin 1A*), *PDI10* (*Protein disulfide isomerase 10*) and *ERO1* (*Endoplasmic reticulum oxidoreductin1*). Data represent means with SEM in both directions among three biological replicates. Each biological replicate contains one shoot rosette.

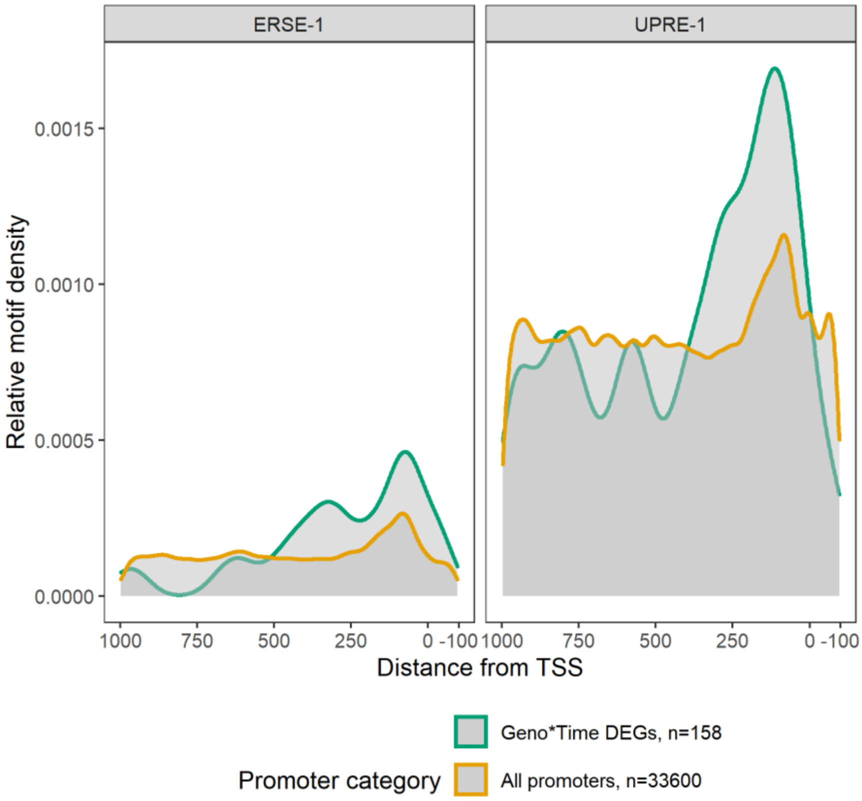

Supplementary figure S14: Relative density of ER-stress related transcriptional motifs ERSE-1 and UPRE-1 (Ko and Brandizzi, 2024). This involved comparison between all promoters (orange line n=33600; non-DEGs) or promoters that show a significant genotype*time interaction effect at any comparison. (n= 158; green lines).

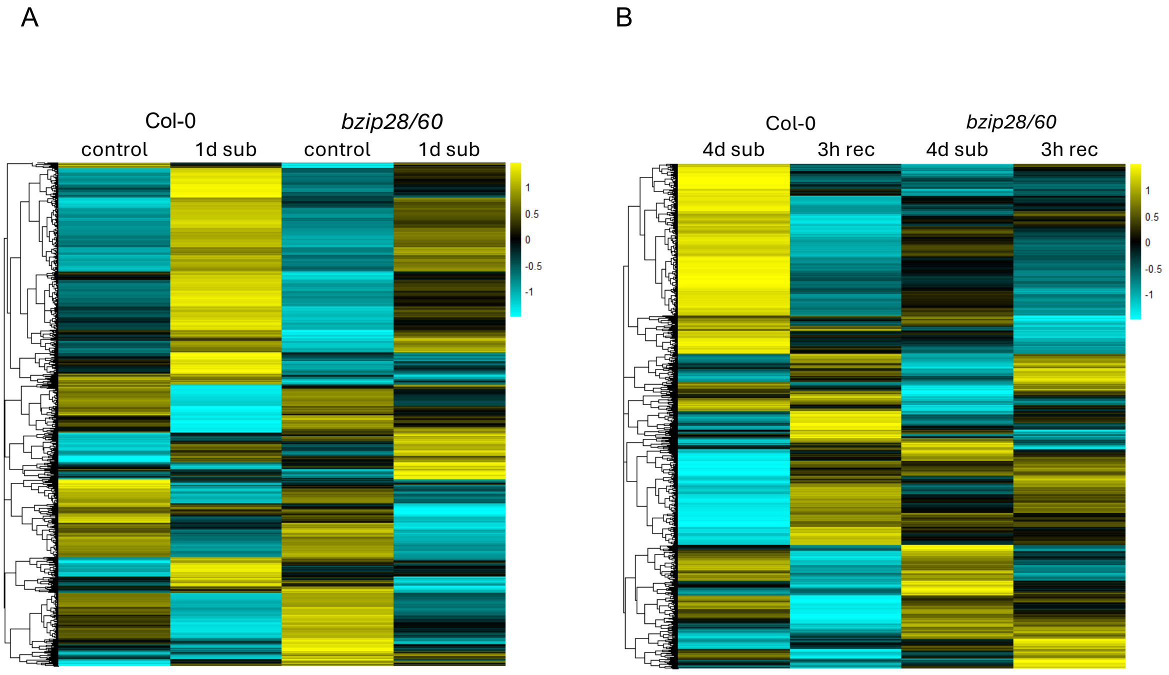

Supplementary Figure S15: Proteome dynamics during submergence and recovery. Heatmaps of scaled (log FC) protein abundance for (A) 3,608 proteins with at least 2 peptides in the 1d submergence experiment; (B) 4,767 proteins with at least 2 peptides in the 4d submergence-3 h recovery experiment.

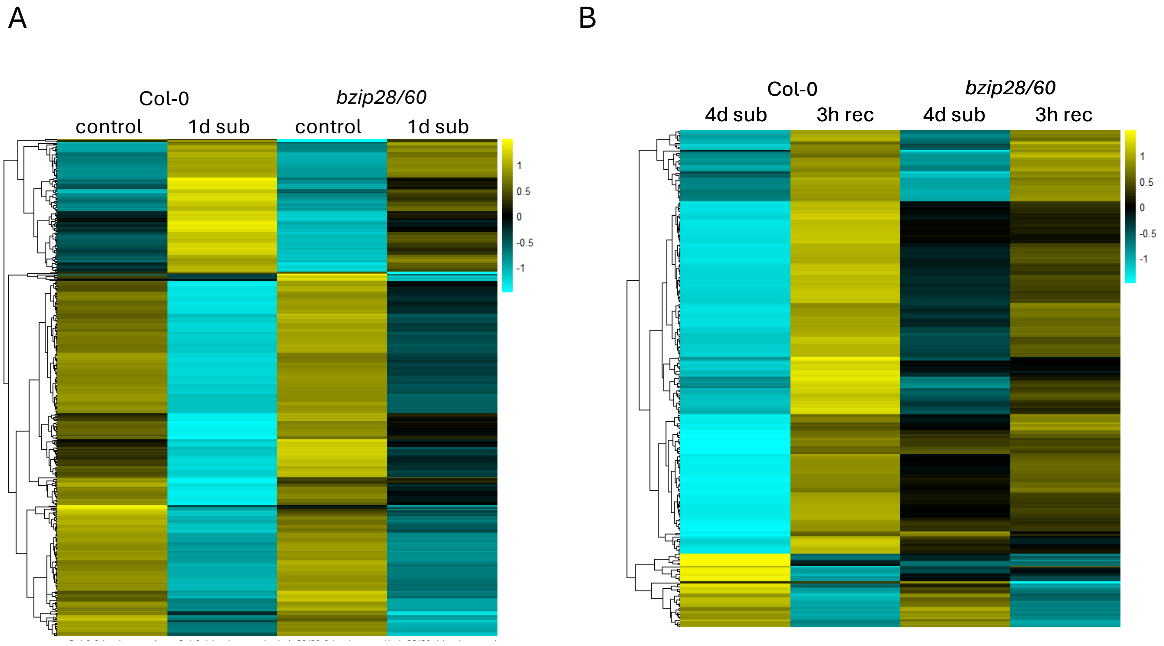

Supplementary Figure S16: The impact of disabled ER stress signalling on the submergence proteome. Heatmaps of scaled (log FC) protein abundance for: (A) 460 proteins with significant 1.5-fold changes ($p < 0.05$) in at least one comparison of Col-0 and *bzip28/60* in the 1d submergence (sub) experiment; (B) 250 proteins with significant 1.5-fold changes ($p < 0.05$) in at least one comparison of Col-0 and *bzip28/60* in the 4d submergence-3 h recovery (rec) experiment.

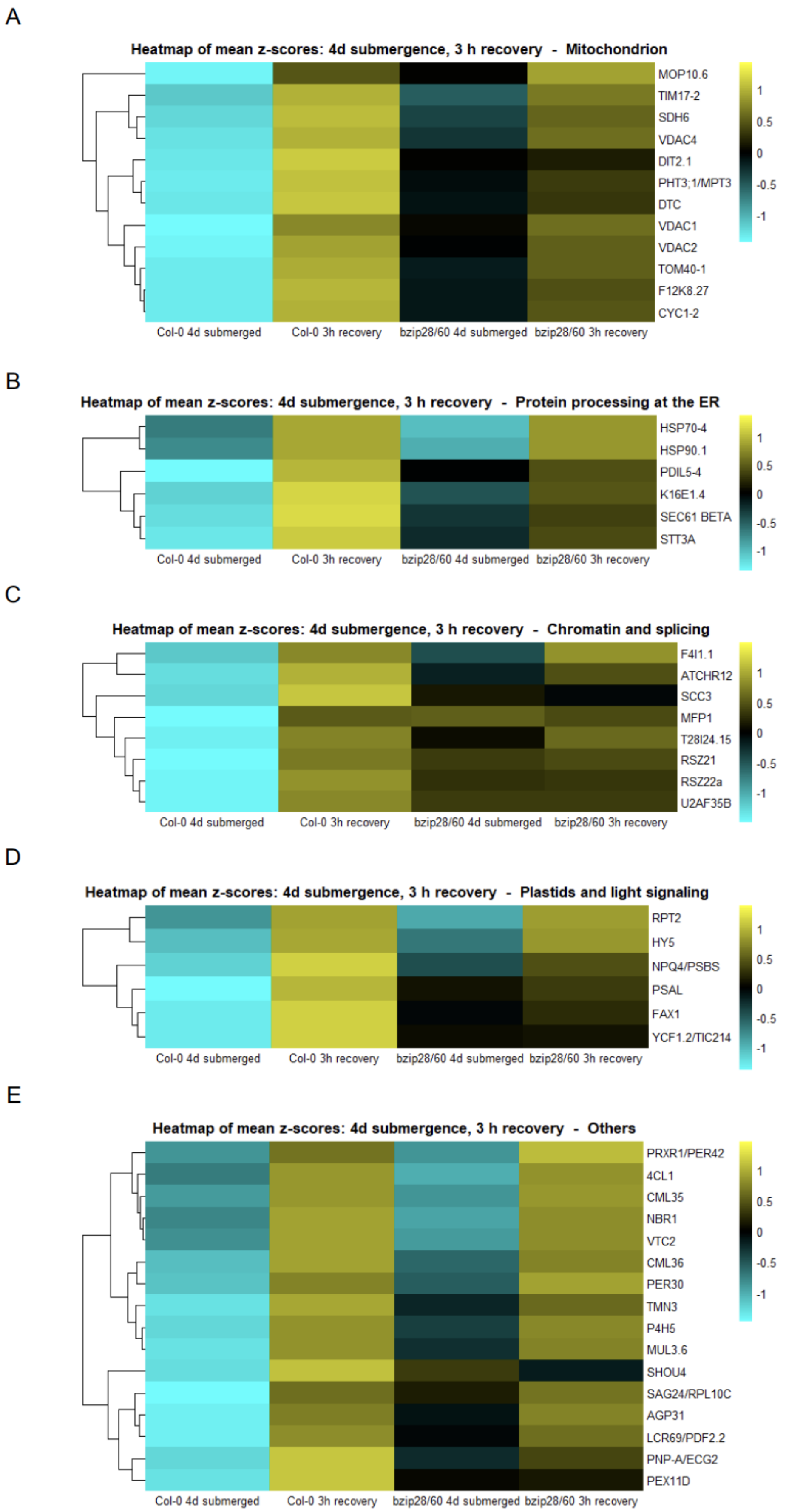
A
Heatmap of mean z-scores: 4d submergence, 3 h recovery - Mitochondrion
MOP10.6
TIM17-2
SDH6
VDAC4
DIT2.1
PHT3;1/MPT3
DTC
VDAC1
VDAC2
TOM40-1
F12K8.27
CYC1-2
Col-0 4d submerged
Col-0 3h recovery
bzip28/60 4d submerged
bzip28/60 3h recovery
1
0.5
0
-0.5
-1
B
Heatmap of mean z-scores: 4d submergence, 3 h recovery - Protein processing at the ER
HSP70-4
HSP90.1
PDIL5-4
K16E1.4
SEC61 BETA
STT3A
Col-0 4d submerged
Col-0 3h recovery
bzip28/60 4d submerged
bzip28/60 3h recovery
1
0.5
0
-0.5
-1
C
Heatmap of mean z-scores: 4d submergence, 3 h recovery - Chromatin and splicing
F4I1.1
ATCHR12
SCC3
MFP1
T28I24.15
RSZ21
RSZ22a
U2AF35B
Col-0 4d submerged
Col-0 3h recovery
bzip28/60 4d submerged
bzip28/60 3h recovery
1
0.5
0
-0.5
-1
D
Heatmap of mean z-scores: 4d submergence, 3 h recovery - Plastids and light signaling
RPT2
HY5
NPQ4/PSBS
PSAL
FAX1
YCF1.2/TIC214
Col-0 4d submerged
Col-0 3h recovery
bzip28/60 4d submerged
bzip28/60 3h recovery
1
0.5
0
-0.5
-1
E
Heatmap of mean z-scores: 4d submergence, 3 h recovery - Others
PRXR1/PER42
4CL1
CML35
NBR1
VTC2
CML36
PER30
TMN3
P4H5
MUL3.6
SHOU4
SAG24/RPL10C
AGP31
LCR69/PDF2.2
PNP-A/ECG2
PEX11D
Col-0 4d submerged
Col-0 3h recovery
bzip28/60 4d submerged
bzip28/60 3h recovery
1
0.5
0
-0.5
-1

Supplementary Figure S17: The impact of disabled ER stress signalling on the submergence proteome. Heatmaps of scaled (log FC) protein abundance for different categories of proteins with increased abundance in Col-0 and *bzip28/60* mutants after 3 h recovery. (A) Mitochondrial proteins; (B) Protein processing at the ER; (C) Chromatin and splicing; (D) Plastids and light signalling; (E) Others.

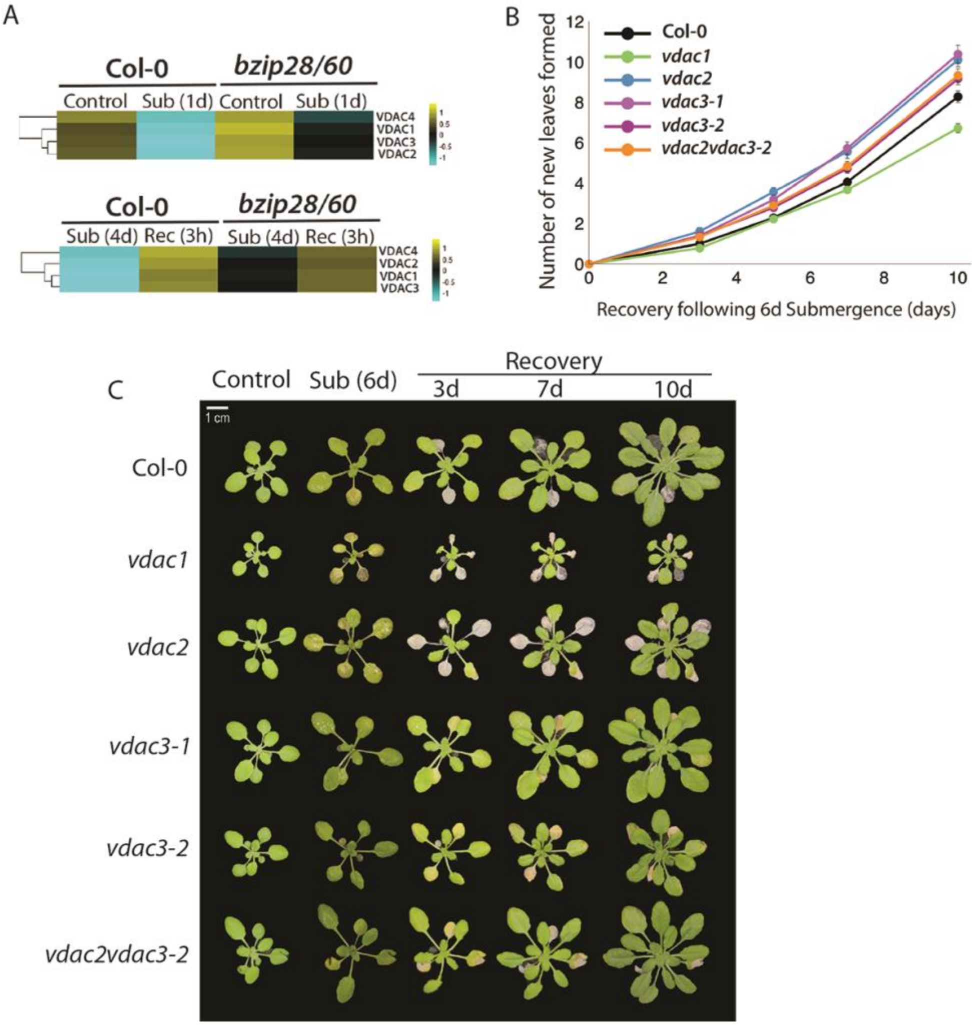

Supplementary Figure S18: Assessment of VDAC contribution to submergence survival. (A) Heatmaps of scaled (log FC) protein abundance for VDAC proteins in Col-0 and *bzip28/60* mutants before (control) and after 1 d submergence (sub) (upper panel); and after 4 d submergence and 3 h post-submergence recovery (rec) (lower panel). (B) Mean number of new leaves formed in Col-0 and *vdac1, vdac2, vdac3-1, vdac3-2* mutants after 6 d submergence, followed by 3, 6, 7 or 10 days of recovery; n=8-10 plants (SE). Statistical significance was calculated using a two-way ANOVA followed by a Tukey's post-hoc test (P <0.05). At the 10d recovery time-point significant differences in new leaf formation were observed for *vdac1*, *vdac2* and *vdac3-1* (See Supplemental Table S3). (C)

Representative images of plants of Col-0 and *vdac* mutants after 6 d of submergence, followed by 3, 7, or 10 d recovery. Scale bar = 1cm. Images were digitally extracted for comparison.

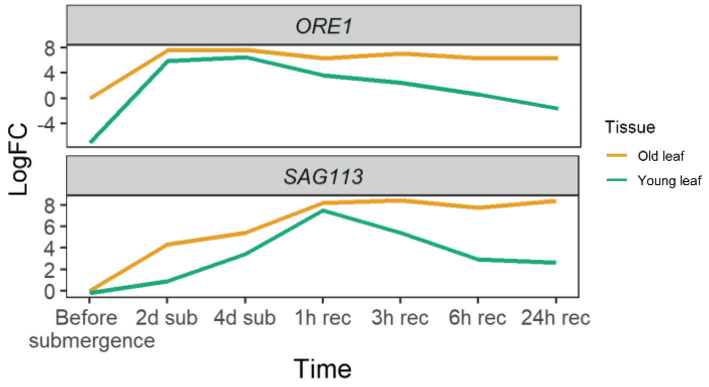

Supplementary figure S19: Expression of *ORE1* and *SAG113* before, during and after submergence. Expression (log FC) was calculated relative to that in old leaves (orange lines) before submergence. Green lines show young leaf expression dynamics (sub = submergence; rec = recovery). Data is extracted for these genes from the transcriptomics dataset where n=3.

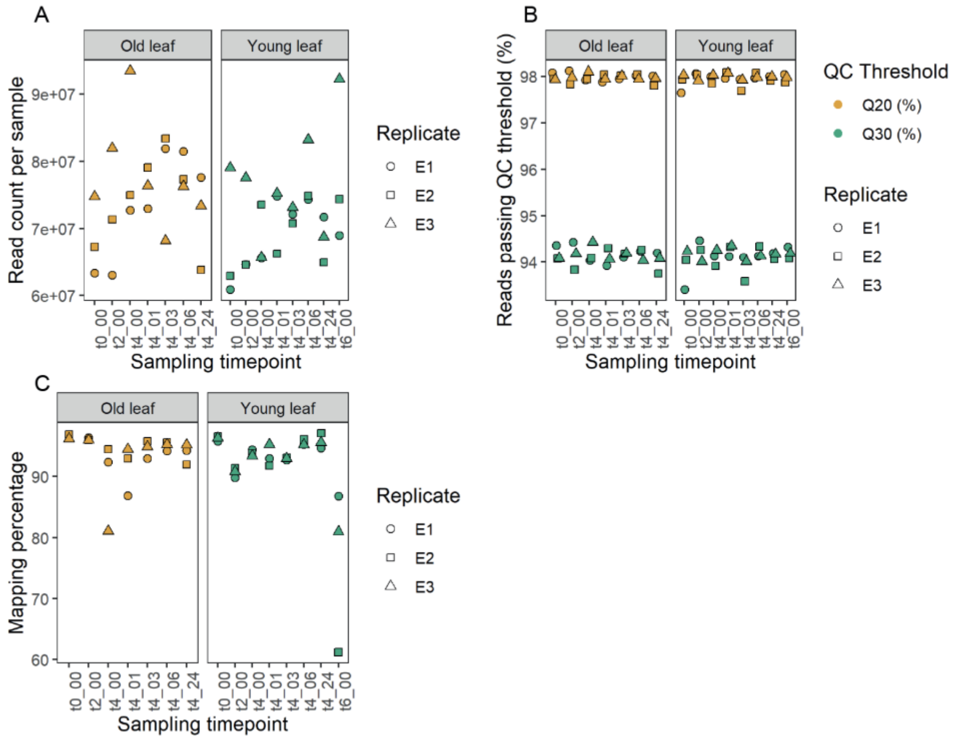

Supplementary figure S20: Quality control of mRNAseq (Col-0 old versus young leaves, see methods)

A) Number of sequenced reads per library of old and young leaves. Shapes indicate different biological replicates

B) Percentage of reads of each library passing the Q20 (99% accuracy) and Q30 (99.9% accuracy) Phred quality thresholds

C) Percentage of reads per library mapped to the Araport11 transcriptome.

A)

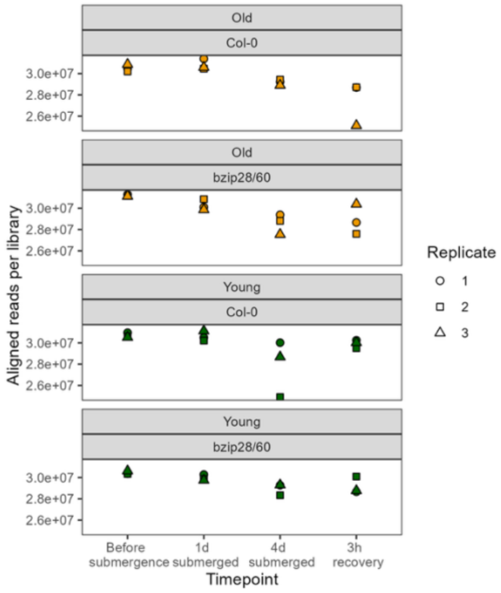

B)

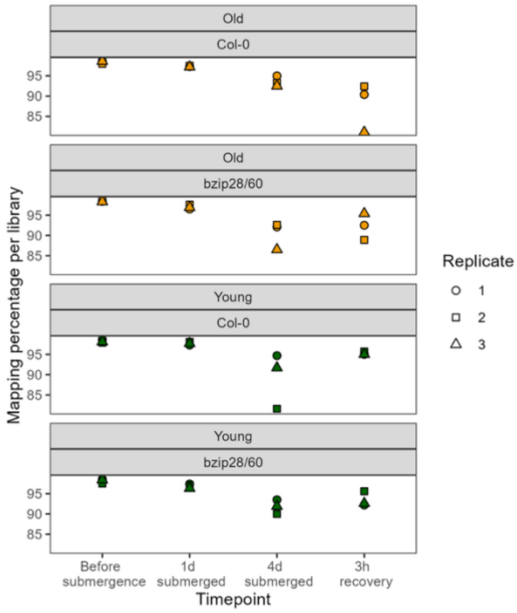

Supplementary figure S21: Quality control of mRNAseq (Col-0 vs *bzip28/60*, old versus young leaves, see methods)

A) Number of sequenced aligned reads per library of old and young leaves. Shapes indicate different biological replicates

B) Percentage of reads per library mapped to the Araport11 transcriptome.

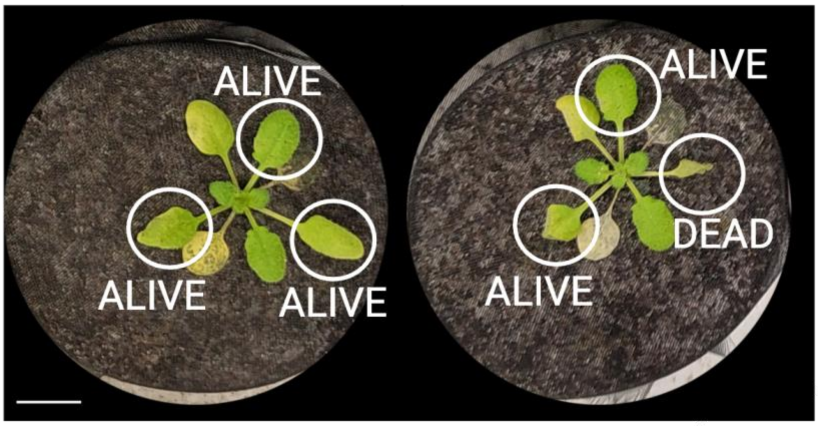

Supplementary figure S22: Scoring for leaf submergence survival. Images showing representative Arabidopsis Col-0 plants that were submerged for varying durations that led to leaf senescence and dehydration. As indicated in the image, for scoring of leaf survival, leaves were scored as dead when approximately more than half of the leaf lamina had desiccated. Scale bar = 1 cm. Scoring was blinded for all survival assays. Images were digitally extracted for comparison. Plants shown in the images are the same plant at different time points and as shown in Figure 1A.
